# Synergistic effects of nitrogen, sewage sludge, and poultry manure on integrative fertilization for wheat and sugar beet in marginally saline soil

**DOI:** 10.3389/fpls.2026.1827328

**Published:** 2026-05-22

**Authors:** Szilvia Veres, Tarek Alshaal, Nevien Elhawat

**Affiliations:** 1 Institute of Applied Plant Biology, Faculty of Agricultural and Food Sciences and Environmental Management, University of Debrecen, Debrecen, Hungary; 2 Department of Food Biotechnology, Albert Kazmer Mosonmagyarovar Faculty, Széchenyi István University, Győr, Hungary; 3 Soil and Water Science Department, Faculty of Agriculture, Kafrelsheikh University, Kafr El-Sheikh, Egypt; 4 Faculty of Agriculture (for Girls), Al-Azhar University, Cairo, Egypt

**Keywords:** biofertilizers, marginally saline soil, organic amendments, soil fertility, soil fertility salinity, sustainable agriculture, wheat and sugar beet

## Abstract

**Introduction:**

Soil salinity is a major constraint to agricultural productivity in arid and semi-arid regions, affecting approximately 1.4 billion hectares globally (over 10% of the world’s land area) and causing substantial yield losses in staple crops, with reductions reaching up to 50% or more in sensitive species under moderate salinity levels.

**Methods:**

While the individual effects of biofertilizers or organic amendments have been widely studied, there remains a critical gap in field-based evidence regarding their synergistic integration—particularly the combined use of dual nitrogen (N_2_)-fixing plant growth-promoting rhizobacteria (*Azotobacter chroococcum* and *Azospirillum lipoferum*) with a balanced blend of sewage sludge and poultry manure (1:1 on N basis) as a 50% substitution for inorganic N in marginally saline soils. This study aimed to evaluate the effects of such integrated fertilization on soil health, nutrient availability, physiological performance, and productivity of wheat (*Triticum aestivum* L. cv. Sakha 93) and sugar beet (*Beta vulgaris* L. cv. Kawemira) in marginally saline soil (initial EC_e_ 3.47 dS/m). A strip-plot field experiment with five treatments was conducted: control (T1), biofertilizer alone (T2), 50% inorganic N + 50% organic manure (T3), biofertilizer + T3 (T4), and conventional NPK (T5). Soil and plant parameters were assessed at 70 days after sowing (DAS), with final yield and quality parameters determined at harvest.

**Results and discussion:**

Treatment T4 significantly improved soil organic matter (23.3 g/kg in wheat and 22.9 g/kg in sugar beet), reduced EC_e_ (to 3.90 dS/m in wheat and 3.13 dS/m in sugar beet), and enhanced available N (up to 63.0 mg/kg) and potassium. It also increased microbial biomass carbon, soil enzyme activities, photosynthetic rate (14.0 µmol CO_2_/m^2^/s in wheat and 26.5 µmol CO_2_/m^2^/s in sugar beet), and alleviated salinity-induced stress indicators. The highest yields were recorded under T4, with wheat grain yield reaching 6719 kg/ha and sugar beet root yield 78.8 tons/ha, accompanied by superior sugar quality. These results demonstrate that the integration of dual PGPR with blended organic amendments (T4) offers a synergistic, low-input strategy that enhances soil fertility, mitigates salinity stress, and boosts crop productivity while reducing inorganic fertilizer use by 50%. This approach provides a scalable and sustainable solution for marginally saline soils in arid regions, supporting food security with a reduced environmental footprint. Further multi-site and multi-year validation across a wider range of salinity levels and soil types is recommended.

## 1 Introduction

Soil salinity is a major global challenge that significantly limits agricultural productivity, particularly in arid and semi-arid regions, by impairing crop growth and yield. The accumulation of soluble salts, such as sodium chloride, in the soil profile reduces water availability, induces osmotic stress and ion toxicity, and disrupts nutrient balance, ultimately affecting plant performance (Abdelrady et al., 2024.; Zombori et al., 2023). Recent global assessments indicate that approximately 1.4 billion hectares (10.7% of the total global land area) are already affected by salinity, with an additional one billion hectares at risk due to climate change and unsustainable land and water management practices. In these salt-affected areas, crop yield reductions commonly range from 20 to 50%, while economic losses associated with salt-induced land degradation in irrigated systems are estimated at approximately US$27.3 billion annually (Qadir et al., 2014).

Staple crops such as wheat (*Triticum aestivum* L.) and sugar beet (*Beta vulgaris* L.) are particularly vulnerable to salinity stress, posing a serious threat to food security due to their importance as cereal and industrial crops, respectively (Alharbi et al., 2022a; Atlason et al., 2015; Baldoni et al., 2023). Wheat, which provides nearly 20% of global caloric intake, is moderately sensitive to salinity, with a threshold of approximately 6 dS/m and a yield reduction rate of about 7.1% per unit increase in salinity beyond this threshold. Under saline conditions, wheat experiences osmotic stress, ion toxicity, and impaired grain filling, particularly during early growth stages (Munns and Tester, 2008). In contrast, sugar beet exhibits relatively higher tolerance to salinity, with a threshold of approximately 7 dS/m; however, salinity still negatively affects root development, sucrose accumulation, and overall biomass production (Wang et al., 2024). These two crops were selected because they represent a typical cereal–root crop rotation system widely practiced in salinity-prone regions (e.g., the Nile Delta), enabling the evaluation of salinity impacts on both food security (wheat) and industrial crop productivity (sugar beet) under integrated soil and water management strategies (Qadir et al., 2014).

Wheat is highly sensitive to salinity, especially during early developmental stages, experiencing osmotic stress that limits water uptake and growth (Alshaal et al., 2024). Prolonged exposure to high salinity leads to ionic stress from excessive sodium (Na^+^) and chloride (Cl^-^) accumulation, disrupting enzymatic functions and causing premature leaf senescence, which significantly reduces yield (Muhammad et al., 2024). Sugar beet, while relatively more tolerant, still suffers from salinity-induced inhibition of root development, reduced sugar content, and decreased biomass (Alharbi et al., 2022a). Salinity affects the osmotic potential of the soil solution and impairs nutrient uptake, particularly potassium (K^+^) and phosphorus (P), which are critical for sugar beet growth and sucrose storage (Khayamim, 2022). These challenges underscore the need for sustainable strategies to mitigate salinity stress and enhance crop productivity in marginally saline soil.

The environmental drawbacks of inorganic fertilizers, including N leaching and greenhouse gas emissions, have shifted focus toward sustainable practices like the use of organic N sources and biofertilizers (Elmeknassi et al., 2024; Zhang et al., 2026b). Poultry manure, rich in N, P, and K^+^, enhances soil physical properties such as water retention and structure while providing a gradual nutrient release that supports crop growth (Arshad et al., 2024). It also boosts microbial activity, facilitating nutrient cycling and organic matter decomposition, which benefits crops like wheat and sugar beet (Hoover et al., 2019). Studies have shown that poultry manure improves wheat grain yield, N uptake, and water use efficiency, while in sugar beet, it enhances root biomass and sugar quality (Jin et al., 2022; Khayamim, 2022).

Organic N sources such as poultry manure and sewage sludge offer sustainable alternatives to inorganic fertilizers. Poultry manure, rich in N, P, and K, enhances soil structure, water retention, and gradual nutrient release while boosting microbial activity, improving wheat grain yield and N uptake, and enhancing sugar beet root biomass and quality (Zuo et al., 2019). Sewage sludge similarly supplies nutrients and organic matter, improving fertility and structure in saline environments, reducing reliance on synthetics, and mitigating leaching (Achkir et al., 2023). Their combined use (1:1 N basis) optimizes C/N ratio, dilutes potential contaminants, and synchronizes nutrient supply, making them particularly suitable for marginally saline soils (Alavilli et al., 2023). Although sewage sludge can contain heavy metals, pharmaceuticals, and pathogens, the material used in the present study was analyzed and showed heavy metal concentrations, e.g., copper (Cu), nickel (Ni), and lead (Pb), were below the permissible limits for agricultural use. Proper treatment and monitoring are essential to minimize potential risks when using sewage sludge as an organic amendment.

Biofertilizers, such as *Azotobacter* sp. and *Azospirillum* sp., offer a sustainable alternative by fixing atmospheric N_2_ and producing growth-promoting substances like phytohormones, siderophores, and enzymes (Vincze et al., 2024). These microorganisms improve nutrient availability and root growth in saline soils, enhancing crop performance (Mahmud et al., 2021). For wheat, biofertilizers increase grain yield and stress tolerance, while in sugar beet, they improve root biomass and sugar content (Raffi and Charyulu, 2021). By reducing the need for chemical fertilizers, biofertilizers minimize environmental risks like nitrate leaching and greenhouse gas emissions, making them cost-effective for saline soil management (Mącik et al., 2020).

The combined use of biofertilizers and organic amendments has shown synergistic effects in enhancing soil health and crop productivity under non-saline conditions (Raffi and Charyulu, 2021). However, that study was conducted on rice using *Azospirillum brasilense* with farmyard manure (FYM) in flooded, non-saline paddies. The present work is the first to evaluate the integrated application of dual PGPR (*Azotobacter chroococcum* + *Azospirillum lipoferum*) combined with a balanced blend of urban (sewage sludge) and animal (poultry manure) wastes on a 1:1 N basis. This integration was tested at 50% substitution of inorganic N in a wheat–sugar beet rotation under field-scale salinity stress (initial EC_e_ = 3.47 dS/m) This integrative approach improves soil structure, microbial activity, and nutrient cycling, reducing reliance on inorganic fertilizers (Hafez et al., 2024). For wheat, it enhances grain protein content and N use efficiency, while for sugar beet, it increases root biomass and sugar yield (Raffi and Charyulu, 2021). Additionally, this strategy improves soil physical properties like porosity and water-holding capacity, critical for crop growth in saline environments (Mahmud et al., 2021). Despite the documented benefits of individual components, no field study has yet evaluated the combined application of dual N_2_-fixing biofertilizers (e.g., *Azotobacter chroococcum* + *Azospirillum lipoferum*) with a balanced blend of urban (sewage sludge) and animal (poultry manure) wastes as 50% inorganic N substitution in a wheat–sugar beet rotation under field-scale salinity stress (initial EC_e_ ≈ 3.5 dS/m). Furthermore, this is the first application of a salinity-weighted Soil Fertility Index (SFI) to quantify improvements from such integrative waste-based fertilization. This research gap limits the development of resilient, low-input strategies for salt-affected agroecosystems.

## 2 Materials and methods

### 2.1 Experimental site

Field experiments were conducted in Kafr El-Sheikh Governorate (30.9396° N, 30.9323° E), Egypt, to evaluate the effects and residual impacts of various agronomic practices on soil quality, plant physiology, and productivity of wheat and sugar beet grown in marginally saline soil. Post-harvest soil samples (0–20 cm) were collected from the previous cotton crop, air-dried, sieved (2 mm), and analyzed for selected physicochemical and microbiological characteristics using standard methods (Sparks et al., 1996). The properties of the experimental soil are detailed in **Supplementary Table 1** (**Supplementary Materials**).

### 2.2 Sources of materials used

#### 2.2.1 Source and characteristics of organic materials

Sewage sludge was sourced from a local sewage treatment plant, and poultry manure was obtained from a private broiler farm. These materials were air-dried, crushed, and sieved (2 mm) before analysis for chemical and microbiological properties (Cottenie, 1980; Jackson, 1973). Their properties are summarized in **Supplementary Table 1** (**Supplementary Materials**).

#### 2.2.2 Source of biofertilizer

The biofertilizer was prepared using PGPR, *Azotobacter chroococcum* SARS 10 and *Azospirillum lipoferum* SP2, mixed in a 1:1 ratio (v/v). These strains were obtained from the Agricultural Research Centre collection and provide the internal identifiers (SARS 10 and SP2) along with the source institution. This ratio was chosen to leverage complementary N_2_-fixation mechanisms: *Azotobacter* fixes atmospheric N_2_ in the bulk soil, while *Azospirillum* promotes associative symbiosis and root proliferation via phytohormone (IAA) production, ensuring balanced microbial activity and enhanced rhizosphere competence under salinity (Vincze et al., 2024). Both strains were pre-screened for salt tolerance: *A. chroococcum* SARS 10, isolated from saline soils (EC_e_ > 6 dS/m), sustains N_2_-fixation up to 8 dS/m, and *A. lipoferum* SP2 maintains growth and hormone synthesis at NaCl levels equivalent to ~10 dS/m (Dobereiner et al., 1976; SWERI strain database). The bacterial strains were provided by the Agricultural Microbiology Department, Soils, Water, and Environment Research Institute (SWERI), ARC, Egypt. The inoculum was prepared following the procedure of Abd-el-Malek et al. (1979) and Dobereiner et al. (1976).

#### 2.2.3 Plant seeds

Wheat seeds (*T. aestivum*, cv. Sakha 93; total duration: 150–160 days from sowing to physiological maturity) and sugar beet seeds (*B. vulgaris*, cv. Kawemira; total duration: 180–200 days from sowing to harvest at 40–45% sugar content) were provided by the Agricultural Research Station, Sakha, and Delta Sugar Co., Al Hamoul, Egypt, respectively. At 70 days after sowing (DAS), wheat was at the late tillering to stem elongation stage (Zadoks 29–39), and sugar beet was in the active leaf and root bulking phase (12–16 leaves, BBCH 39), both representing peak vegetative growth and nutrient demand under salinity stress (Alharbi et al., 2022a; El-Hendawy et al., 2017).

#### 2.2.4 Source of applied inorganic NPK

Nitrogen was supplied as ammonium nitrate (33.5% N), (P) as calcium superphosphate (15.5% P_2_O_5_), and (K) as potassium sulfate (50% K_2_SO_4_).

### 2.3 Treatments

The experiment included five treatments (**Table 1**) applied to wheat and sugar beet: i) T1: Control (no fertilizers or amendments), ii) T2: Biofertilizer inoculation (*A. chroococcum* SARS 10 and *A. lipoferum* SP2) + recommended inorganic P and K, iii) T3: 50% inorganic N and 50% organic manure (sewage sludge and poultry manure in a 1:1 ratio based on N content) + recommended P and K, iv) T4: Biofertilizer + T3, and v) T5: Conventional inorganic NPK fertilizers. The 50% inorganic + 50% organic N substitution in T3/T4 was selected to: (1) synchronize rapid (inorganic) and slow-release (organic) N supply, minimizing leaching in saline soils; (2) optimize C/N ratio (~6.7) via 1:1 blending of sewage sludge (C/N 6.27) and poultry manure (C/N 7.14) for sustained microbial activity; (3) dilute heavy metals (e.g., Pb reduced from 14.0 to ~8.8 mg/kg per unit N); and (4) achieve maximum inorganic N reduction without yield loss, as 50% is the upper threshold for reliable mineralization under salinity stress (Alavilli et al., 2023; Hafez et al., 2024). The recommended inorganic NPK were calculated per hectare per crop (wheat and sugar beet) according to the recommendations from the Ministry of Agriculture and Soil Reclamation, Egypt.

**Table 1:** Description of applied treatments and time of application in wheat and sugar beet plantations in marginally saline soil.

Crop	Applied fertilizer/manure	T1		T2		T3		T4		T5	
		Amount (kg/ha)	Time	Amount (kg/ha)	Time	Mount (kg/ha)	Time	Amount (kg/ha)	Time	Amount (kg/ha)	Time
Wheat	N (N)	-	–	–	–	80	-16 kg/ha with soil preparation -32 kg/ha at 30 DAS^†^ (tillering stage) -32 kg/ha at 60 DAS (stem elongation stage)	80	-16 kg/ha with soil preparation -32 kg/ha at 30 DAS (tillering stage) -32 kg/ha at 60 DAS (stem elongation stage)	160	-16 kg/ha with soil preparation -32 kg/ha at 30 DAS (tillering stage) -32 kg/ha at 60 DAS (stem elongation stage)
	P (P_2_O_5_)	-	-	60	with soil preparation	60	with soil preparation	60	with soil preparation	60	with soil preparation
	K (K_2_SO_4_)	-	-	40	-10 kg/ha with soil preparation -30kg/ha 30 DAS	40	-10 kg/ha with soil preparation -30kg/ha 30 DAS	40	-10 kg/ha with soil preparation -30kg/ha 30 DAS	40	-10 kg/ha with soil preparation -30kg/ha 30 DAS
	Sewage sludge^‡^	-	-	-	-	3000	with soil preparation	3000	with soil preparation	-	-
	Poultry manure	-	-	-	-	3000	with soil preparation	3000	with soil preparation	-	-
	Biofertilizer	–	–	1.4	at seed sowing	–	–	1.4	at seed sowing	–	–
Sugar beet	N (N)	-	-	-	-	100	-20kg/ha with soil preparation -40kg/ha at 30DAS (early growth stage) -40kg/ha at 51DAS (root bulking stage)	100	-20kg/ha with soil preparation -40kg/ha at 30DAS (early growth stage) -40kg/ha at 51DAS (root bulking stage)	200	-20kg/ha with soil preparation -40kg/ha at 30DAS (early growth stage) -40kg/ha at 51DAS (root bulking stage)
	P (P_2_O_5_)	-	-	80	with soil preparation	80	with soil preparation	80	with soil preparation	80	with soil preparation
	K (K_2_SO_4_)	-	-	100	-50 kg/ha with soil preparation -50 kg/ha 51 DAS	100	-50 kg/ha with soil preparation -50 kg/ha 51 DAS	100	-50 kg/ha with soil preparation -50 kg/ha 51 DAS	100	-50 kg/ha with soil preparation -50 kg/ha 51 DAS
	Sewage sludge	-	-	-	-	7500	with soil preparation	7500	with soil preparation	-	-
	Poultry manure	-	-	-	-	7500	with soil preparation	7500	with soil preparation	-	-
	Biofertilizer	–	–	1.4	at seed sowing	–	–	1.4	at seed sowing	–	–

T1: Control (no fertilizers or amendments), ii) T2: Biofertilizer inoculation (*Azotobacter chroococcum* SARS 10 and *Azospirillum lipoferum* SP2) + recommended inorganic P and K, iii) T3: 50% inorganic N and 50% organic manure (sewage sludge and poultry manure in a 1:1 ratio based on N content) + recommended P and K, iv) T4: Biofertilizer + T3, and v) T5: Conventional inorganic NPK fertilizers.

^†^ DAS day after sowing.

^‡^Organic manure in T3 and T4: sewage sludge + poultry manure (1:1 N basis), blended to optimize C/N, reduce metal risk, and ensure synchronized N release.

### 2.4 Experimental arrangement

The experiments were arranged in a strip-plot design with two crops (wheat and sugar beet) as row factors and five treatments (T1–T5) as column factors. Each treatment combination was replicated three times. Individual plots measured 2 × 3 m and were separated by 1 m-wide ditches. Wheat and sugar beet were sown on November 14^th^ at seed rates of 143 and 12 kg/ha, respectively. Plant spacing, irrigation, and agronomic practices followed the national recommendations for wheat and sugar beet production in Egypt (Ministry of Agriculture and Land Reclamation, 2022). After the sugar beet seeds germinated, the plants were thinned to one plant per hill. Details of the applied treatments, including the amounts and timing of application, are summarized in **Table 1**. The microbial inoculum was prepared by incubating *Azotobacter chroococcum* SARS 10 and *Azospirillum lipoferum* SP2 for two days at 30 °C. A peat-based inoculum was then created by mixing 1 mL of each bacterial suspension (10^8^ CFU/mL) with 1 gram of peat. Wheat and sugar beet seeds were spread on a clean plastic sheet in the shade and mixed with the peat-based inoculum at a rate of 1.4 kg/ha just before sowing.

### 2.5 Soil sampling and analysis

Soil samples were collected at 70 days after sowing (DAS) from a depth of 0–20 cm in each experimental plot. Three subsamples were taken from each plot (three replicate plots per treatment) and combined to form one representative composite sample per plot. Plant debris, stones, and gravel were removed, after which the composite samples were air-dried at room temperature, ground, and passed through a 2 mm sieve for chemical analysis. For microbial and biochemical analyses, additional soil samples were collected simultaneously from the same plots, placed in sterilized polyethylene bags, transported to the laboratory in an icebox, sieved through an 8 mm mesh, and stored at −20 °C until analysis. All laboratory analyses were performed in triplicate. Soil pH was measured in a 1:2.5 soil-to-water suspension using a pH meter (Genway 3510, Cambridgeshire, UK), while electrical conductivity (EC_e_) was determined from soil paste extracts using an EC meter (Jenway 4310, Cambridgeshire, UK) as described by Sparks et al. (1996). Organic matter (OM) was quantified via wet combustion using the potassium dichromate oxidation method (Sparks et al., 1996). Cation exchange capacity (CEC) was evaluated through the sodium acetate saturation technique (Sparks et al., 1996).

The soil fertility index (SFI) was calculated to evaluate the impact of fertilization treatments on soil health and productivity, following the methodology of Qi et al. (2009). The SFI incorporates key soil parameters such as pH, EC_e_, soil organic matter (SOM), CEC, available nutrients (N, P, and K), total nutrient content (N and P), and trace metals (Cu, Ni, and Pb). The specific formula and weight factors were applied according to the reference methodology.


$$\text{SFI}=\sum_{i=1}^{n}\left(\frac{observed\;value\;of\;soil\;parameter\;i}{Optimum\;value\;of\;soil\;parameter\;i}\right)\times weight\;of\;soil\;parameter\;i.$$

where n is the total number of indicators, if no weights are given equal weights of parameters is assumed.

Available N (Ava-N) was extracted using 2M KCl and analyzed following the semi-micro Kjeldahl method as described by Sparks et al. (1996). Available P (Ava-P) was extracted using 0.5 M sodium bicarbonate (Olsen et al., 1954) and quantified colorimetrically using the ascorbic acid method (Sparks et al., 1996). Available K (Ava-K) was extracted with ammonium acetate, following the method of Cottenie (1980), and measured using Atomic Absorption Spectrophotometry (AAS, PerkinElmer 3300, Shelton, Connecticut, USA)) (Jackson, 1973).

Total N (Total-N) content was determined in digested soil samples using a mixture of H_2_SO_4_ and HClO_4_ (10:3 v/v) and analyzed using the semi-micro Kjeldahl method (Sparks et al., 19). Total P (Total-P) was measured in digested samples prepared with a mixture of HNO_3_ and HClO_4_ (10:3 v/v) and quantified using AAS (PerkinElmer 3300, Shelton, USA) (Jackson, 1973).

Extractable soil Cu, Pb, and Ni were extracted using DTPA (diethylene triamine penta acetic acid) and quantified with AAS (PerkinElmer 3300, Shelton, Connecticut, USA.) following the protocol outlined by Jackson (1973). DTPA-extractable Cu, Pb, and Ni were quantified to assess plant-available fractions under salinity stress, where metal solubility may increase despite high soil pH (Zuo et al., 2019). The other metals were not analyzed following pre-treatment screening showing concentrations below the detection limits in both sewage sludge and poultry manure, confirming negligible contamination risk.

Microbial biomass C (MBC; mg/kg soil) was assessed using the chloroform fumigation-extraction procedure (Brookes et al., 1985). In this method, 20.0 g of soil was fumigated with ethanol-free chloroform for 24 h. Both fumigated and non-fumigated samples were extracted with 80 mL of 0.5 M K_2_SO_4_ by shaking for 30 min on a reciprocating shaker at 40 rpm, followed by filtration. Carbon content in the extracts was determined using an Elementar Vario Max Cube analyzer (Elementar Analysensysteme GmbH, Germany).

Glucose-induced soil respiration was measured in thawed soil samples incubated at 35 °C for 10 days after the addition of 80 mg glucose/g soil. After incubation, respiration was determined over 24 h using NaOH to capture released CO_2_ (Ölinger et al., 1996). Soil moisture content was adjusted to 60% prior to incubation.

Microbiological analyses of soil samples followed the protocol of Louw and Webley (1959). The serial dilution pipette method was used to quantify microbial populations on selective media: total bacterial count on Thornton’s medium (Allen, 1952), total fungal count on Martin’s medium (Allen, 1952), total actinomycetes count on Jensen’s medium (Allen, 1952), *Azotobacter* spp. on modified Ashby’s medium (Abd-el-Malek et al., 1979), and *Azospirillum* spp. on N-deficient semi-solid medium (Dobereiner et al., 1976). It should be noted that the serial dilution plate count method enumerates only culturable microorganisms, which represent a small fraction of the total soil microbial community.

Soil dehydrogenase activity was determined spectrophotometrically using the 2,3,5-triphenyl tetrazolium chloride (TTC) method (3% w/v) as described by Chander and Brookes (1991). Absorbance was measured at 485 nm, and a standard curve was prepared using triphenyl formazan (TPF). Soil phosphatase activity was measured using p-nitrophenyl phosphate as a substrate, with absorbance recorded at 440 nm, following the method of Dick et al. (2000). Catalase activity was quantified using the titration method proposed by Johnson and Temple (1964). Invertase activity was evaluated using the Schaffer-Somogyi micro method (Vitolo and Borzani, 1983), which quantifies the reducing sugars released after soil incubation with a 5% sucrose solution. Absorbance was recorded with a UV-160A spectrophotometer (Shimadzu, Kyoto, Japan).

### 2.6 Plant sampling and determination of plant-related parameters

All the following plant-related analyses were done in triplicate. Seventy days after seed sowing, the youngest fully expanded leaves from the tips of wheat and sugar beet plants were collected to assess physiological and biochemical stress markers. Relative water content (RWC) was measured using 1 cm^2^ leaf discs. Fresh mass (FM) of the discs was initially recorded, after which the discs were submerged in distilled water for 5 h to determine the turgid mass (TM). The discs were then dried at 70 °C for 48 h to obtain the dry mass (DM). RWC was calculated following the formula outlined by Barrs and Weatherley (1962).


$$RWC\;\left(\%\right)=\frac{FM-DM\;}{TM-DM}\times 100.$$

Electrolyte leakage (EL) was determined using ten leaf discs, each with an area of 1 cm^2^. The discs were placed in a test tube containing 10 mL of distilled water and incubated in a water bath at 55 °C for 25 min. The initial electrical conductivity (EC1) was then measured. The samples were subsequently heated to 100 °C for an additional 10 min to measure the second electrical conductivity (EC2). EL was calculated following the procedure described by (Bajji et al. (2002).


$$EL\;\left(\%\right)=\frac{EC1}{EC2}\;\times 100.$$

Malondialdehyde (MDA) content, a marker of lipid peroxidation in the phospholipid bayer, was measured from 0.5 g of fresh leaves ground in liquid N_2_. The thiobarbituric acid (TBA) method, as outlined by Du and Bramlage (1992), was used. Absorbance of the resulting solution was recorded at 532 nm and 600 nm using a UV-160A spectrophotometer (Shimadzu, Kyoto, Japan).

Proline content was determined from 500 mg of fresh leaves ground in 3% H_2_SO_4_. The homogenate was centrifuged at 12,000 rpm for 5 min, and the supernatant was mixed with toluene. Proline concentration was quantified using ninhydrin reagent following the method described by Bates et al. (1973). Absorbance was measured at 520 nm using a UV-160A spectrophotometer (Shimadzu, Kyoto, Japan).

F N:P:K analysis in plant tissues, 0.5 g of ground material was digested in a Kjeldahl tube with 5 mL of sulfuric acid (H_2_SO_4_). The digestion process involved gradually increasing the temperature by 5 °C per min until reaching 170 °C, where it was maintained for 2 h. After cooling for 30 min, 2 mL of 30% H_2_O_2_ was added, and the temperature was raised to 120 °C for another h until the solution cleared. The digested sample volume was adjusted to 50 mL with ultra-pure water in a volumetric flask. The N content was analyzed using the conventional Kjeldahl method (Sparks et al., 1996), while P and K contents were determined using an Atomic Absorption Spectrophotometer (AAS, PerkinElmer 3300, Shelton, Connecticut, USA) with a detection limit of 100 ppb, according to Cottenie (1980).

Photosynthetic pigments were evaluated 80 days after seed sowing following the method described by Lichtenthaler (1987). Briefly, 1 g of fresh leaf tissue was homogenized in 6 mL of 80% acetone. The samples were incubated overnight in the dark at room temperature and subsequently centrifuged at 12,000 rpm for 15 min. Absorbance of the supernatant was recorded at 645 nm, 663 nm, and 470 nm using a UV-160A spectrophotometer (Shimadzu, Kyoto, Japan). The concentrations of chlorophyll a, chlorophyll b, and carotenoids (µg/g FW) were calculated using the following formulas:


$$\begin{array}{l} \text{Chlorophyll a}=12.7\left({\text{A}}_{663}\right)-2.69\left({\text{A}}_{645}\right)\\ \text{Chlorophyll b}=25.8\left({\text{A}}_{645}\right)-4.68\left({\text{A}}_{663}\right)\\ \text{Carotenoids}=\left(1000\left({\text{A}}_{470}\right)-2.27\left(\text{chl a}\right)-81.4\left(\text{chl b}\right)\right)/227 \end{array}$$

Photosynthetic rate was measured in the youngest fully expanded leaf 80 days after seed sowing. The evaluation was conducted under optimal conditions with a light intensity of 1000 μmol/m^2^/s using the LI-6400 portable photosynthesis system (Li-COR, Lincoln, NE, USA) at a temperature of 30 ± 2 °C. The CO_2_ concentration ranged from 350 to 400 μmol/mol, and the vapor pressure deficit (VPD) was adjusted to align with 50% relative humidity (RH), following the protocol by Tian-gen et al. (2017).

At the experiment’s conclusion (April 18^th^ for wheat and May 5^th^ for sugar beet), ten wheat plants per plot were randomly harvested to measure FM and DM (g/plant), plant height (cm), spike length (cm), grains per spike, and the weight of 1000 grains (g). Grain yield (kg/ha) was calculated for the entire plot area. Similarly, ten sugar beet plants per plot were harvested to assess root fresh mass (g/plant), shoot fresh mass (g/plant), leaf length (cm), leaf width (cm), root length (cm), root width (cm), root/shoot ratio, sugar content (%), sugar quality (%), root K content (mmol/100g), root sodium content (mmol/100g), and α-amino content (mmol/100g). Root and sugar yields were calculated for the total plot area. Sugar content and quality of sugar beet roots were analyzed at the Al Hamoul laboratory, Delta Sugar Co., Kafr El-Sheikh, Egypt.

The contribution rate of fertilizers (CRF; %) for wheat and sugar beet yields was calculated to assess the effects of different fertilizer treatments on crop productivity using the formula:


$$\text{CRF}=\frac{\left(Y\;fertilizer-Y\;control\right)}{Y\;fertilizer}\times 100$$

(Ou et al., 2018)

where Y control is yield in the control plot (ton/ha) and Y fertilizer is yield in the fertilized plot (ton/ha).

### 2.7 Bioassay trial

A bioassay trial was conducted to evaluate the residual effects of the applied treatments (T1–T5). Soil samples (0–20 cm) were collected from the experimental plots at harvest and used to germinate and grow soybean (*Glycine max* L.) plants. Briefly, glass test tubes (30 × 4 cm) were filled with 50 g of air-dried soil. Five soybean seeds were sown in each tube and covered with a thin layer (1–2 cm) of sterilized washed sand. The tubes were then irrigated to approximately field capacity using sterilized distilled water. Prior to sowing, the seeds were sterilized by immersion in 50% H_2_SO_4_ for 2 min, followed by thorough washing with sterile distilled water until a neutral pH was achieved. After one week, the seedlings were thinned to two plants per tube. The experiment followed a randomized complete block design with nine replicates per treatment. Whole soybean plants were harvested 21 days after sowing, and FM, DM, and N:P:K content were determined.

### 2.8 Statistical analysis

Data were analyzed using a strip-plot (split-block) design with crops (wheat, sugar beet) as row (strip) factors and fertilization treatments (T1–T5) as column factors, with three replicates per treatment-crop combination. All variables were checked for normality (Shapiro-Wilk test) and homogeneity of variance (Levene’s test).Two-way ANOVA was performed to evaluate main effects of crop, treatment, and their interaction (crop × treatment). When interactions were significant (P ≤ 0.05), one-way ANOVA was conducted separately for each crop, followed by Tukey’s *post-hoc* test at α = 0.05 to compare treatment means within each crop. Soil fertility index (SFI) and microbial counts were log-transformed prior to analysis to meet ANOVA assumptions. All statistical analyses were conducted using IBM SPSS Statistics v.27.0 (IBM Corp., Armonk, NY, USA). Treatment effects were considered significant at *P* ≤ 0.05, *P* ≤ 0.01, and *P* ≤ 0.001.

## 3 Results

### 3.1 Response of soil chemical properties to organic and biofertilizers

Soil pH remained relatively stable, with only minor variations observed (**Table 2**). Under wheat, the highest pH was recorded in T2 (8.19 ± 0.02), followed by T3 (8.18 ± 0.03) and T1 (8.16 ± 0.02), while T4 had the lowest (8.13 ± 0.03). Under sugar beet, T1 recorded the highest pH (8.27 ± 0.03), with T4 again showing the lowest (8.20± 0.03). The T4 treatment consistently reduced soil pH relative to the highest-pH treatment in each crop. This slight decrease in pH under T4 indicates a potential acidifying effect, likely attributed to the use of biofertilizers and organic amendments. EC_e_ showed a more pronounced reduction in sugar beet compared to wheat. The lowest EC_e_ for sugar beet (3.13 dS/m) was observed in T2, which also resulted in the second-lowest EC_e_ for wheat (3.90 dS/m). T4 also demonstrated significant reductions in EC_e_, while T5 exhibited the highest EC_e_ values for both crops (**Table 2**). The SOM increased across all treatments compared to the control (T1), with the most notable improvements under T2 and T4. Sugar beet under T2 and T4 saw SOM increases of 22.4 g/kg and 22.9 g/kg, respectively, while wheat experienced increases of 23.8 g/kg and 23.3 g/kg under the same treatments. The T4 achieved the highest CEC values for wheat (45.9 cmolc/kg) and sugar beet (40.0 cmolc/kg), indicating enhanced nutrient retention. Similarly, the SFI followed this trend, with the highest values recorded under T4 for sugar beet (0.98) and wheat (0.886).

**Table 2:** Response of different soil properties to different agronomic practices (T1-T5)† and different vegetative cover [wheat (*Triticum aestivum* L., cv. Sakha 93) and sugar beet (*Beta vulgaris* L., cv. Kawemira)] in marginally saline soil.

Property	T1		T2		T3		T4		T5	
	Wheat	Sugar beet	Wheat	Sugar beet	Wheat	Sugar beet	Wheat	Sugar beet	Wheat	Sugar beet
pH	8.16 ± 0.03b	8.27 ± 0.02a	8.19 ± 0.03ab	8.24 ± 0.03a	8.18 ± 0.03ab	8.25 ± 0.03a	8.13 ± 0.03d	8.20 ± 0.03b	8.17 ± 0.01b	8.26 ± 0.02a
ECe^‡^	4.28 ± 0.15b	2.36 ± 0.05f	3.90 ± 0.12c	3.13 ± 0.10e	4.61 ± 0.18a	3.24 ± 0.10d	4.37 ± 0.17b	2.53 ± 0.07f	4.67 ± 0.18a	3.30 ± 0.10d
SOM^¥^ (g/kg)	22.9 ± 0.40b	20.8 ± 0.20d	23.8 ± 0.41a	22.4 ± 0.30c	23.0 ± 0.40ab	22.9 ± 0.30b	23.3 ± 0.41a	22.9 ± 0.30b	23.3 ± 0.20a	22.8 ± 0.50b
CEC[Table-fn fnT2_4]	34.2 ± 0.50d	35.7 ± 0.40c	35.8 ± 0.53c	36.8 ± 0.50c	41.2 ± 0.59b	39.5 ± 0.50b	45.9 ± 0.65a	40.0 ± 0.50b	39.0 ± 0.28b	39.3 ± 0.50b
SFI^*^	0.81 ± 0.03d	0.93 ± 0.02b	0.84 ± 0.04c	0.95 ± 0.02a	0.84 ± 0.04c	0.98 ± 0.02a	0.89 ± 0.05b	0.98 ± 0.03a	0.85 ± 0.03c	0.97 ± 0.02a
Ava-N (mg/kg)	13.8 ± 1.32e	32.7 ± 2.00c	14.0 ± 1.34e	42.0 ± 3.00b	16.4 ± 1.56d	56.0 ± 2.50a	16.4 ± 1.56d	63.0 ± 2.50a	15.2 ± 0.72d	58.0 ± 2.00a
Ava-P (mg/kg)	4.7 ± 0.19b	4.2 ± 0.30c	5.6 ± 0.24a	5.3 ± 0.40b	5.0 ± 0.34b	4.7 ± 0.30b	6.8 ± 0.14a	6.8 ± 0.50a	5.5 ± 0.46b	4.5 ± 0.30c
Ava-K (mg/kg)	73.3 ± 1.02b	59.5 ± 2.00d	89.2 ± 1.24a	61.7 ± 2.50c	75.5 ± 1.05b	61.4 ± 2.50c	97.9 ± 1.36a	80.4 ± 3.00b	84.0 ± 2.82a	61.5 ± 3.00c
Total-N (g/kg)	1.03 ± 0.05b	0.76 ± 0.02d	1.05 ± 0.05b	0.92 ± 0.03c	1.20 ± 0.05a	0.96 ± 0.03b	1.23 ± 0.06a	1.04 ± 0.04b	1.13 ± 0.05a	0.97 ± 0.02b
Total- P (g/kg)	0.09 ± 0.01c	0.11 ± 0.00b	0.11 ± 0.01b	0.15 ± 0.00a	0.11 ± 0.00b	0.16 ± 0.01a	0.14 ± 0.00a	0.16 ± 0.01a	0.11 ± 0.00b	0.16 ± 0.01a

^†^ T1: Control (no fertilizers or amendments), ii) T2: Biofertilizer inoculation (*Azotobacter chroococcum* SARS 10 and *Azospirillum lipoferum* SP2) + recommended inorganic P and K, iii) T3: 50% inorganic N and 50% organic manure (sewage sludge and poultry manure in a 1:1 ratio based on N content) + recommended P and K, iv) T4: Biofertilizer + T3, and v) T5: Conventional inorganic NPK fertilizers. The recommended inorganic NPK were calculated per hectare per each crop (wheat and sugar beet) according to the recommendations from the Ministry of Agriculture and Soil Reclamation, Egypt.

^‡^ Electrical conductivity of soil solution (dS/m).

^¥^ Soil organic matter.

**^
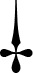
^ :**Cation exchange capacity of soil (cmole_c_/kg).

^*^ Soil fertility index.

Means in the same row of the same crop plant followed by different letters are significant according to the Tukey’s test at *p* ≤ 0.05. Data are means ± SD. (n=3).

### 3.2 Changes in soil nutrient contents

The Ava-N increased under all treatments (**Table 2**), with the highest values in T4 (16.4 mg/kg for wheat and 63.0 mg/kg for sugar beet). Sugar beet consistently exhibited higher N availability, particularly in T4 and T3. The Ava-P was highest in T4 for both crops (6.8 mg/kg), emphasizing the positive impact of biofertilizers combined with organic amendments. Moreover, Ava-P levels were consistently lower in sugar beet than in wheat, reflecting crop-specific nutrient dynamics. Similarly, Ava-K peaked in T4 for wheat (97.9 mg/kg) and sugar beet (80.4 mg/kg), showcasing the benefits of integrated nutrient management. For Total-N, T4 demonstrated the highest values for wheat (1.23 g/kg) and sugar beet (1.04 g/kg). The Total-P followed a similar pattern, with T4 achieving the highest contents for both crops. Sugar beet exhibited consistently higher Total-P levels than wheat, indicating its stronger affinity for phosphorus accumulation.

### 3.3 Changes in soil MBC and respiration

**Figure 1:**
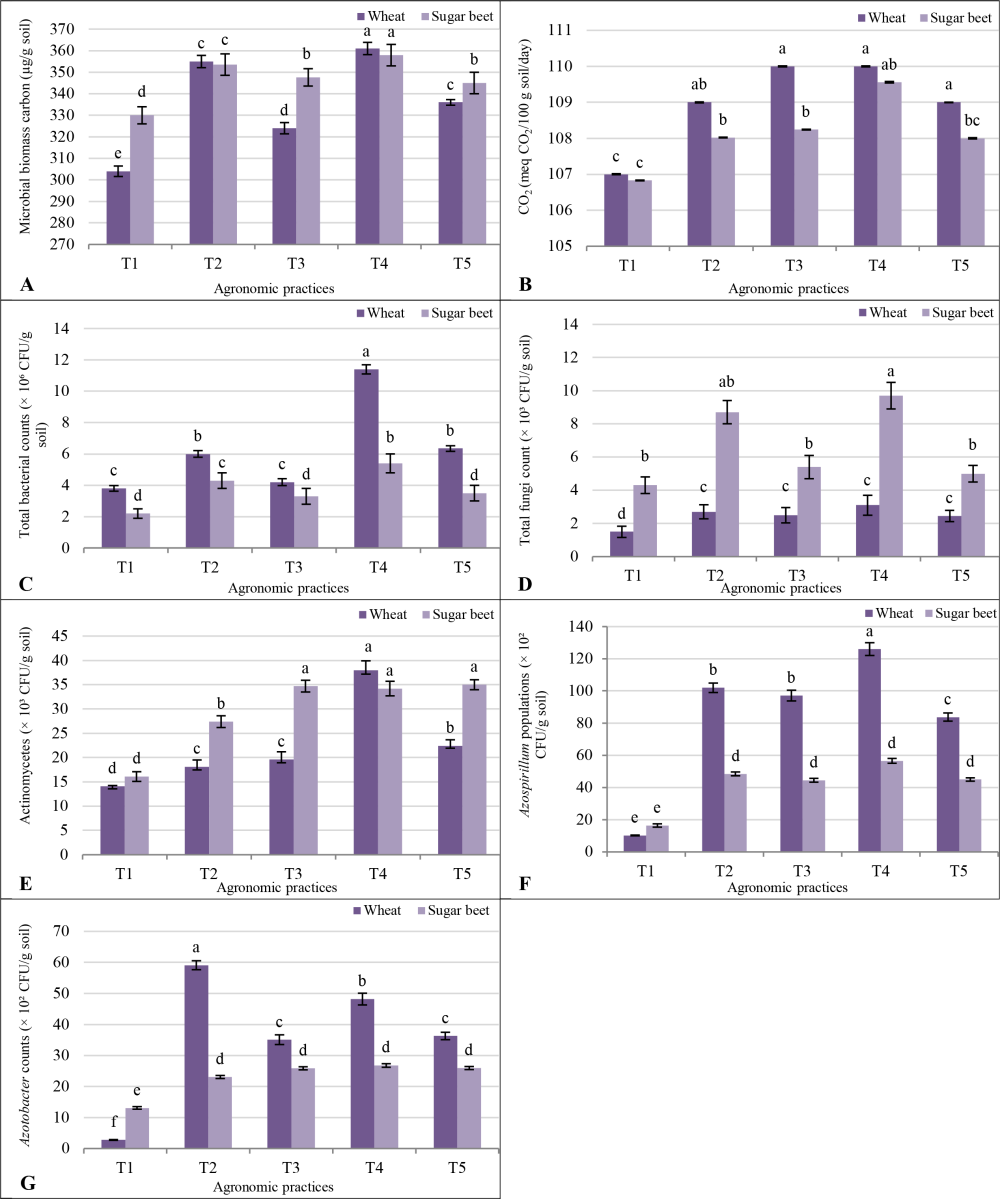
Response of soil microbial biomass carbon (MBC) **(A)**; soil respiration (CO_2_) **(B)**; total microbial counts **(C-G)** to different agronomic practices (T1-T5) and different vegetative cover (wheat (*Triticum aestivum* L., cv. Sakha 93) and sugar beet (*Beta vulgaris* L., cv. Kawemira)) grown in marginally saline soil. T1: Control (no fertilizers or amendments), ii) T2: Biofertilizer inoculation (*Azotobacter chroococcum* SARS 10 and *Azospirillum lipoferum* SP2) + recommended inorganic P and K, iii) T3: 50% inorganic N and 50% organic manure (sewage sludge and poultry manure in a 1:1 ratio based on N content) + recommended P and K, iv) T4: Biofertilizer + T3, and v) T5: Conventional inorganic NPK fertilizers. The recommended inorganic NPK were calculated per hectare per each crop (wheat and sugar beet) according to the recommendations from the Ministry of Agriculture and Soil Reclamation, Egypt. Different letters on bars are significant according to the Tukey’s test at *p* ≤ 0.05. Data are means ± SD. (n=3).

Wheat plants in T1 showed an MBC of 304 µg/g soil, while sugar beet had a slightly higher value of 330 µg/g soil (**Figure 1**). The T2 boosted MBC for both crops, with wheat reaching 355 µg/g soil and sugar beet 354 µg/g soil, reflecting the significance of inoculation with PGPRs. T4 further increased MBC to 361 µg/g soil in wheat and 358 µg/g soil in sugar beet. T3 resulted in moderate MBC increases (324 µg/g soil for wheat and 348 µg/g soil for sugar beet), while T5 showed lower MBC levels (336 µg/g soil for wheat and 345 µg/g soil for sugar beet). The T1 recorded 107 meq CO_2_/100 g soil/day for both crops (**Figure 1**). Treatment T2 slightly increased emissions to 109 for wheat and 108 for sugar beet, while T4 showed the highest rates at 110 for both crops. The T3 resulted in emissions of 110 for wheat and 108 for sugar beet. The T5 maintained consistent levels at 109 for wheat and 108 for sugar beet.

### 3.4 Changes in soil microbial counts

In the T1, total bacterial counts (× 10^6^ CFU/g soil) were higher in wheat (3.8) than in sugar beet (2.2). The T2 significantly increased counts for both crops, with wheat rising to 6.0 and sugar beet to 4.3 (**Figure 1**). The treatment T4 showed the most pronounced effect, with wheat reaching 11.4 and sugar beet 5.4. The treatment T3 saw a decline compared to T4 but remained above the control (4.2 for wheat and 3.3 for sugar beet). The T5 resulted in moderate counts, slightly higher for wheat (6.4) than sugar beet (3.5). Fungal populations (× 10³ CFU/g soil) were consistently higher in sugar beet. The T1 recorded 4.3 for sugar beet and 1.5 for wheat. The treatment T2 doubled these counts to 2.7 in wheat and 8.7 in sugar beet. The treatment T4 achieved the highest counts, with wheat at 3.1 and sugar beet at 9.7. T3 and T5 showed reduced fungal activity, though sugar beet maintained higher counts than wheat. Actinomycetes (× 10³ CFU/g soil) followed a similar trend, with sugar beet consistently outperforming wheat (**Figure 1**). The T1 recorded 14.1 for wheat and 16.1 for sugar beet. The T4 treatment saw the largest increase, reaching 38.0 in wheat and 34.2 in sugar beet. The T3 and T5 resulted in moderate increases, with sugar beet maintaining higher counts. *Azotobacter* counts (× 10^2^ CFU/g soil) were higher in sugar beet under T1 (13.1) than in wheat (2.8). Treatments T2 and T4 significantly boosted populations, with wheat reaching 59.1 and sugar beet 23.1 under T2, and even higher under T4 (48.2 in wheat and 26.8 in sugar beet) (**Figure 1**). *Azospirillum* populations (× 10^2^ CFU/g soil) mirrored this trend. The T1 treatment showed higher counts in sugar beet (16.4) than in wheat (10). The T4 resulted in the highest counts, with wheat at 126 and sugar beet at 56.5. Treatments T3 and T5 showed reduced but still notable increases compared to T1 (**Figure 1**).

### 3.5 Changes in soil enzyme activities

**Figure 2:**
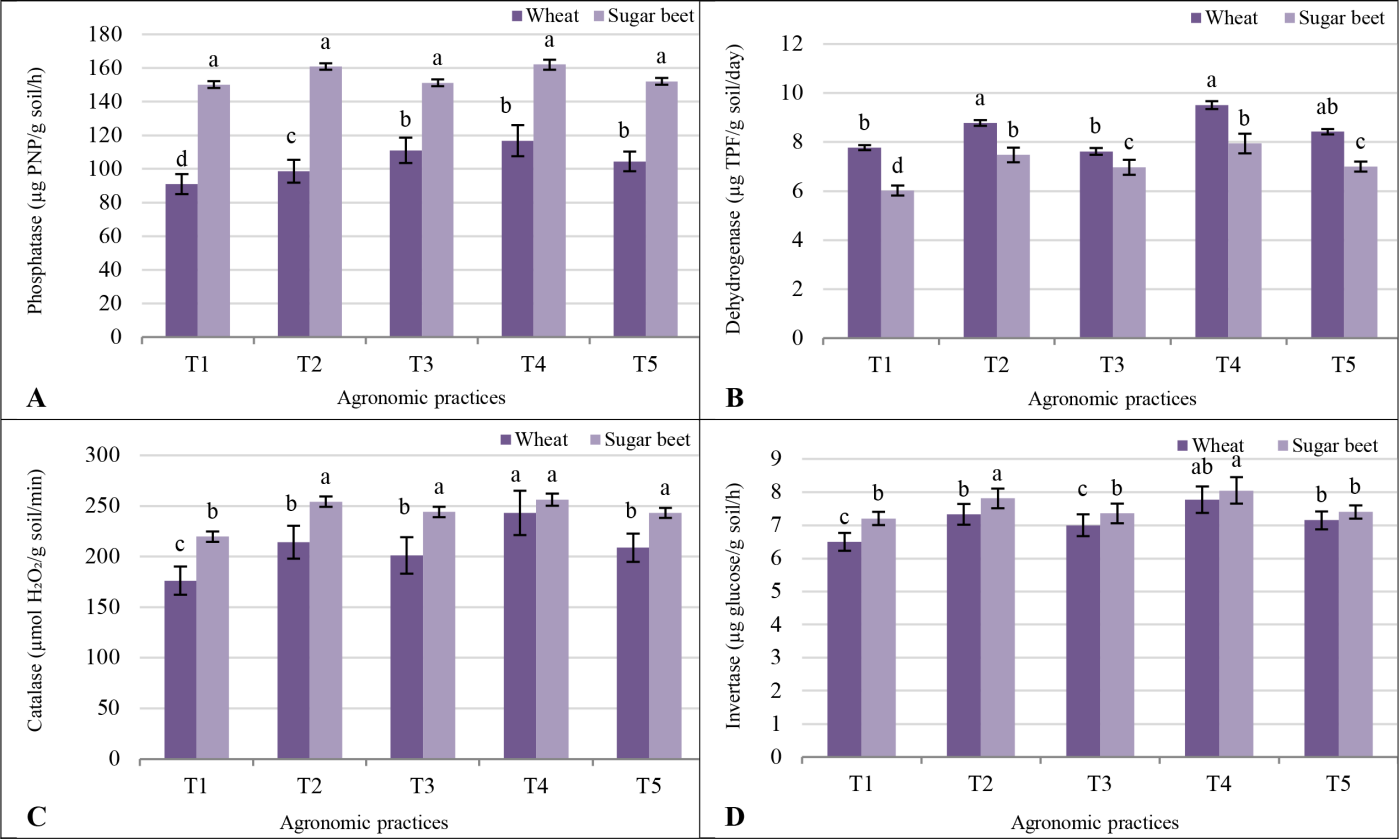
Response of soil enzyme activities **(A)** phosphatase; **(B)** dehydrogenase; **(C)** catalase; and **(D)** invertase to different agronomic practices (T1-T5) and different vegetative cover (wheat (*Triticum aestivum* L., cv. Sakha 93) and sugar beet (*Beta vulgaris* L., cv. Kawemira)) grown in marginally saline soil. T1: Control (no fertilizers or amendments), ii) T2: Biofertilizer inoculation (*Azotobacter chroococcum* SARS 10 and *Azospirillum lipoferum* SP2) + recommended inorganic P and K, iii) T3: 50% inorganic N and 50% organic manure (sewage sludge and poultry manure in a 1:1 ratio based on N content) + recommended P and K, iv) T4: Biofertilizer + T3, and v) T5: Conventional inorganic NPK fertilizers. The recommended inorganic NPK were calculated per hectare per each crop (wheat and sugar beet) according to the recommendations from the Ministry of Agriculture and Soil Reclamation, Egypt. Different letters on bars are significant according to the Tukey’s test at *p* ≤ 0.05. Data are means ± SD. (n=3).

Phosphatase activity was higher in soils cultivated with sugar beet than wheat across all treatments, reflecting sugar beet’s greater influence on phosphorus cycling. Under the control treatment (T1), phosphatase activity was 91 µg PNP/g soil/h for wheat and 150.1 µg PNP/g soil/h for sugar beet (**Figure 2**). The T2 enhanced phosphatase activity in both crops, with wheat increasing to 98.7 µg PNP/g soil/h and sugar beet reaching 160.9 µg PNP/g soil/h. Treatment T4 resulted in the highest phosphatase activity, particularly in wheat (116.8 µg PNP/g soil/h), while sugar beet showed a slight increase (162.0 µg PNP/g soil/h). Treatments T3 and T5 also improved phosphatase activity compared to T1, but the increases were more pronounced in sugar beet than wheat. The T3 reached 111.1 µg PNP/g soil/h for wheat and 151.2 µg PNP/g soil/h for sugar beet, while T5 resulted in 104.4 µg PNP/g soil/h and 152.0 µg PNP/g soil/h, respectively.

Dehydrogenase activity, indicative of microbial metabolic activity, was generally higher in wheat soils. In T1, activity was 7.77 µg TPF/g soil/day for wheat and 6.02 µg TPF/g soil/day for sugar beet. The T2 increased dehydrogenase activity to 8.78 µg TPF/g soil/day for wheat and 7.47 µg TPF/g soil/day for sugar beet. Treatment T4 resulted in the highest values for wheat (9.51 µg TPF/g soil/day) and sugar beet (7.94 µg TPF/g soil/day), demonstrating the additive benefits of co-organic manure and biofertilizers. In T3 and T5, dehydrogenase activity slightly decreased but remained higher than T1, with wheat consistently outperforming sugar beet (**Figure 2**).

Catalase activity, which indicates oxidative stress mitigation in soil, was consistently higher in sugar beet soils. In T1, activity was 176 µmol H_2_O_2_/g soil/min for wheat and 220 µmol H_2_O_2_/g soil/min for sugar beet. The T2 significantly enhanced catalase activity, reaching 214 µmol H_2_O_2_/g soil/min for wheat and 254 µmol H_2_O_2_/g soil/min for sugar beet. Treatment T4 exhibited the highest catalase activity, with wheat reaching 243 µmol H_2_O_2_/g soil/min and sugar beet 256 µmol H_2_O_2_/g soil/min. Under T3 and T5, catalase activity remained elevated compared to T1, though sugar beet consistently outperformed wheat across all treatments (**Figure 2**).

Invertase activity, which is crucial for carbohydrate metabolism, showed smaller differences between crops and treatments. In treatment T1, activity was 6.5 µg glucose/g soil/h for wheat and 7.21 µg glucose/g soil/h for sugar beet. Treatment T2 increased invertase activity to 7.3 µg glucose/g soil/h for wheat and 7.81 µg glucose/g soil/h for sugar beet. Treatment T4 again yielded the highest invertase activity for both crops, with wheat at 7.8 µg glucose/g soil/h and sugar beet at 8.05 µg glucose/g soil/h. Treatments T3 and T5 showed moderate increases over T1, with wheat values reaching 7.0 µg glucose/g soil/h and 7.2 µg glucose/g soil/h, respectively, and sugar beet values at 7.36 µg glucose/g soil/h and 7.4 µg glucose/g soil/h (**Figure 2**).

### 3.6 Stress indicators

**Figure 3:**
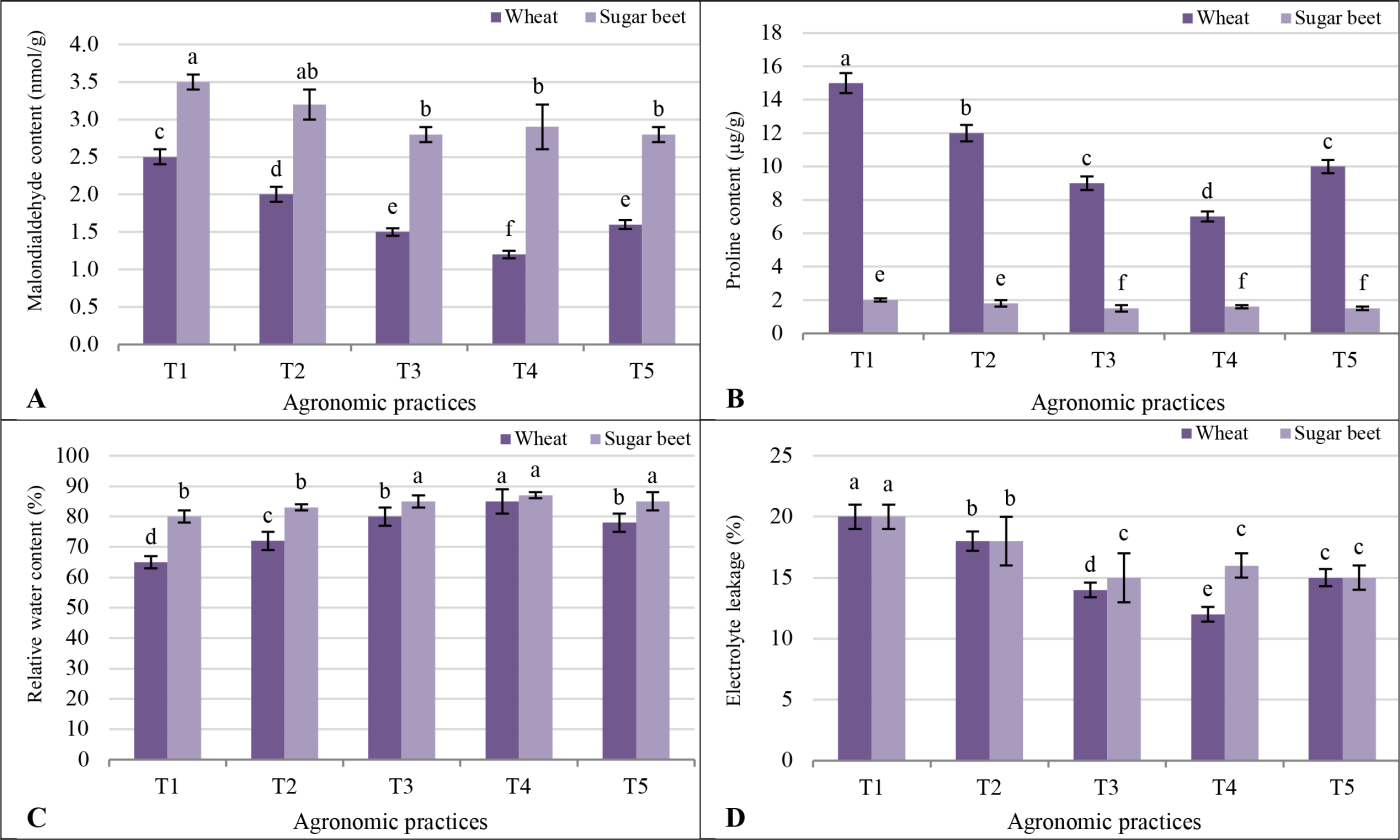
Response of different stress indicators **(A)** malondialdehyde content; **(B)** proline content; **(C)** relative water content; and **(D)** electrolyte leakage to different agronomic practices (T1-T5) and different vegetative cover [wheat (*Triticum aestivum* L., cv. Sakha 93) and sugar beet (*Beta vulgaris* L., cv. Kawemira)] grown in marginally saline soil. T1: Control (no fertilizers or amendments), ii) T2: Biofertilizer inoculation (*Azotobacter chroococcum* SARS 10 and *Azospirillum lipoferum* SP2) + recommended inorganic P and K, iii) T3: 50% inorganic N and 50% organic manure (sewage sludge and poultry manure in a 1:1 ratio based on N content) + recommended P and K, iv) T4: Biofertilizer + T3, and v) T5: Conventional inorganic NPK fertilizers. The recommended inorganic NPK were calculated per hectare per each crop (wheat and sugar beet) according to the recommendations from the Ministry of Agriculture and Soil Reclamation, Egypt. Different letters on bars are significant according to the Tukey’s test at *p* ≤ 0.05. Data are means ± SD. (n=3).

The MDA content, an indicator of lipid peroxidation and oxidative stress, was generally higher in sugar beet than wheat across all treatments (**Figure 3**). The T4 treatment resulted in the lowest MDA content for both crops, with wheat recording 1.2 nmol/g and sugar beet 2.9 nmol/g. Treatments T3 and T5, which involved co-organic and inorganic fertilization, showed slightly higher MDA levels compared to T4 but significantly lower than T1 (control), which exhibited the highest MDA content in both wheat (2.5 nmol/g) and sugar beet (3.5 nmol/g). Proline content, a marker of osmotic adjustment under stress, was significantly higher in wheat than sugar beet in all treatments, indicating greater proline accumulation in wheat as a salinity response. The T4 treatment showed the most pronounced reduction in proline for both crops, with wheat and sugar beet recording 7.0 µg/g and 1.6 µg/g, respectively. The control treatment (T1) led to the highest proline levels in wheat (15.0 µg/g) and sugar beet (2.0 µg/g), reflecting higher stress levels under inadequate nutrient support (**Figure 3**). The RWC, a measure of plant water status, was consistently higher in sugar beet than wheat across treatments, suggesting better water retention in sugar beet. The T4 treatment maximized RWC in both crops, with wheat reaching 85% and sugar beet 87%. T3 and T5 also enhanced RWC compared to T1, but to a slightly lesser extent. The control treatment recorded the lowest RWC, with wheat at 65% and sugar beet at 80% (**Figure 3**). The EL, indicative of membrane stability, was generally comparable between wheat and sugar beet, though sugar beet showed slightly lower leakage under effective treatments. The T4 treatment resulted in the least EL for both crops (12% for wheat and 16% for sugar beet). T3 and T5 followed, with slightly higher EL but still significantly better than T1, where wheat and sugar beet exhibited the highest EL (20%) (**Figure 3**).

### 3.7 Photosynthesis efficiency

**Figure 4:**
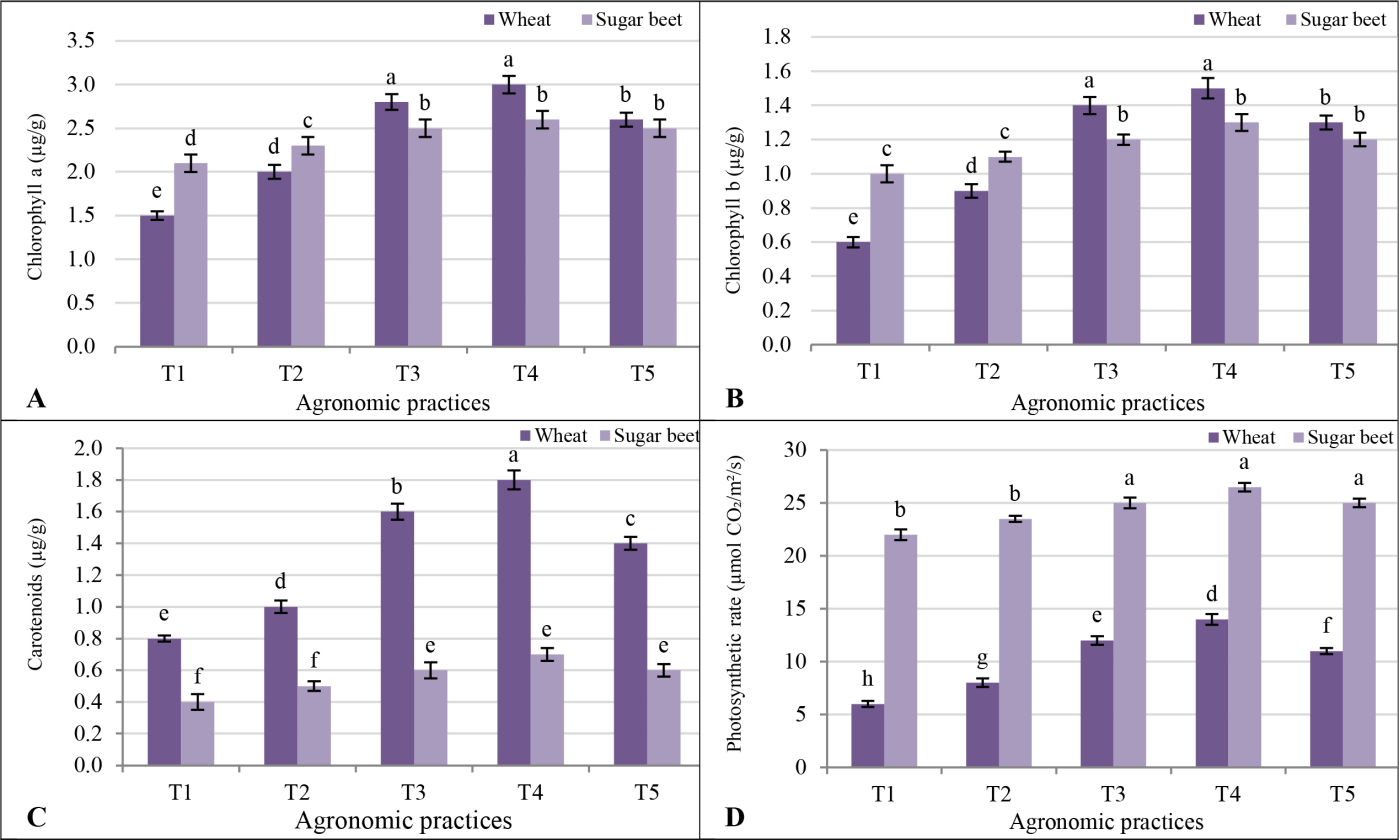
Response of photosynthetic machinery **(A)** chlorophyll a; **(B)** chlorophyll a; **(C)** carotenoids; and **(D)** photosynthetic rate to different agronomic practices (T1-T5) and different vegetative cover (wheat (*Triticum aestivum* L., cv. Sakha 93) and sugar beet (*Beta vulgaris* L., cv. Kawemira)) grown in marginally saline soil. T1: Control (no fertilizers or amendments), ii) T2: Biofertilizer inoculation (*Azotobacter chroococcum* SARS 10 and *Azospirillum lipoferum* SP2) + recommended inorganic P and K, iii) T3: 50% inorganic N and 50% organic manure (sewage sludge and poultry manure in a 1:1 ratio based on N content) + recommended P and K, iv) T4: Biofertilizer + T3, and v) T5: Conventional inorganic NPK fertilizers. The recommended inorganic NPK were calculated per hectare per each crop (wheat and sugar beet) according to the recommendations from the Ministry of Agriculture and Soil Reclamation, Egypt. Different letters on bars are significant according to the Tukey’s test at *p* ≤ 0.05. Data are means ± SD. (n=3).

For chlorophyll, wheat consistently had lower levels compared to sugar beet across all treatments. Among the treatments, T4 recorded the highest chlorophyll a in wheat (3.0 µg/g) and sugar beet (2.6 µg/g). The treatment T3 showed slightly reduced values for both crops. The control treatment (T1) exhibited the lowest chlorophyll a for both crops (**Figure 4**). Chlorophyll b followed a similar trend, with higher values in sugar beet than wheat. Again, T4 emerged as the most effective treatment, with wheat recording 1.5 µg/g and sugar beet 1.3 µg/g, compared to significantly lower values under T1 (0.6 µg/g for wheat and 1.0 µg/g for sugar beet). The T4 provided a synergistic effect, enhancing chlorophyll b in both crops compared to individual treatments or inorganic fertilizers alone (**Figure 4**). In terms of carotenoids, wheat showed consistently higher levels than sugar beet across all treatments. Treatment 4 significantly increased carotenoid content in wheat to 1.8 µg/g and sugar beet to 0.7 µg/g. Treatments T3 and T5 also enhanced carotenoids compared to T1 but to a lesser extent than T4, underscoring the contribution of biofertilizers (**Figure 4**). The photosynthetic rate exhibited stark differences between wheat and sugar beet, with sugar beet outperforming wheat in all treatments. The highest rates were observed under T4 for both crops, where wheat achieved 14.0 µmol CO_2_/m^2^/s and sugar beet 26.5 µmol CO_2_/m^2^/s. Treatments T3 and T5 followed, though with slightly reduced rates compared to T4, emphasizing the comparative advantage of integrating biofertilizers.

### 3.8 Plant NPK contents

**Figure 5:**
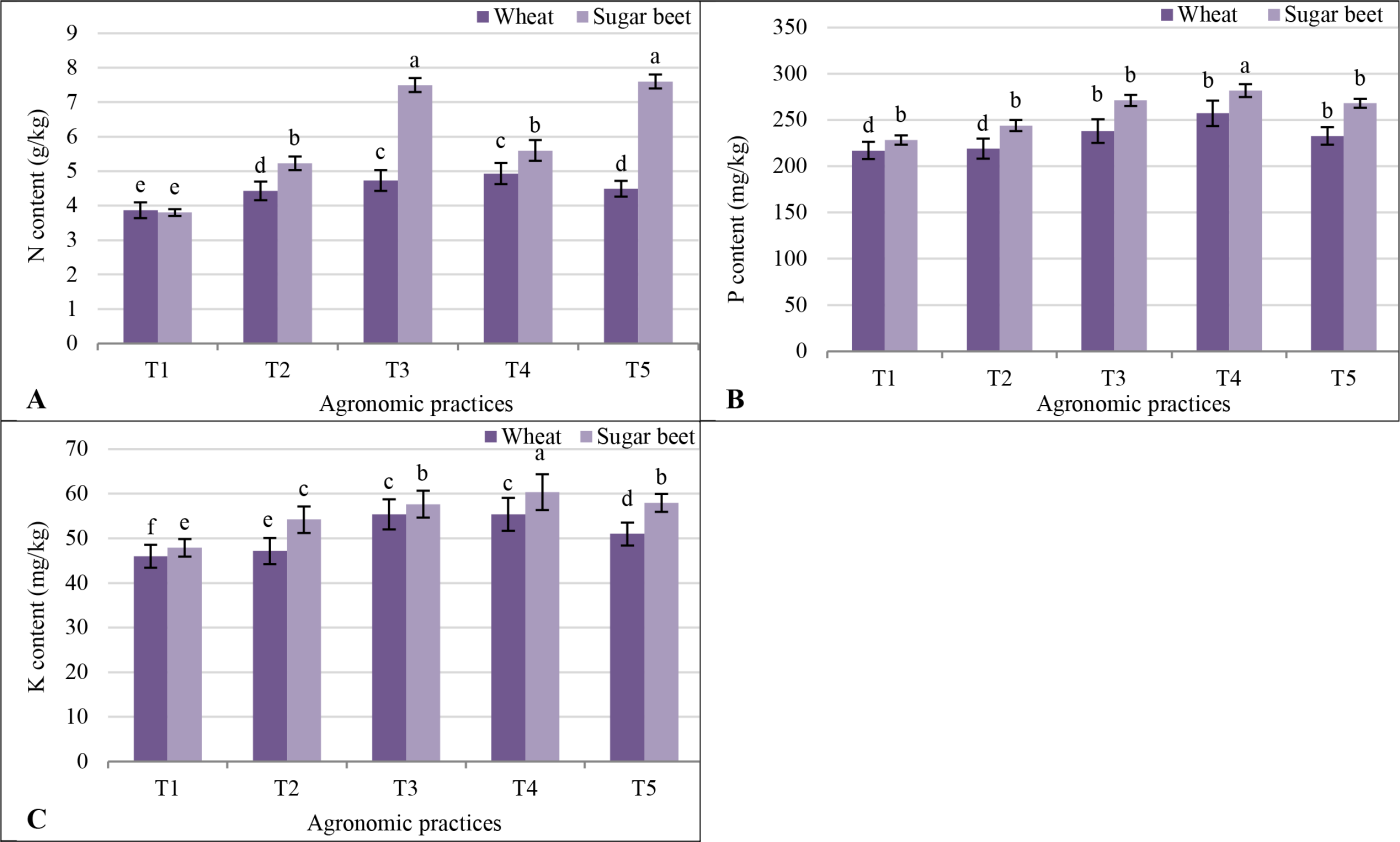
Response of plant NPK **(A)** N content; **(B)** P content; and **(C)** K content to different agronomic practices (T1-T5) and different vegetative cover [wheat (*Triticum aestivum* L., cv. Sakha 93) and sugar beet (*Beta vulgaris* L., cv. Kawemira)] grown in marginally saline soil. T1: Control (no fertilizers or amendments), ii) T2: Biofertilizer inoculation (*Azotobacter chroococcum* SARS 10 and *Azospirillum lipoferum* SP2) + recommended inorganic P and K, iii) T3: 50% inorganic N and 50% organic manure (sewage sludge and poultry manure in a 1:1 ratio based on N content) + recommended P and K, iv) T4: Biofertilizer + T3, and v) T5: Conventional inorganic NPK fertilizers. The recommended inorganic NPK were calculated per hectare per each crop (wheat and sugar beet) according to the recommendations from the Ministry of Agriculture and Soil Reclamation, Egypt. Different letters on bars are significant according to the Tukey’s test at *p* ≤ 0.05. Data are means ± SD. (n=3).

Plant NPK contents were highest under T4, with sugar beet consistently outperforming wheat. T4 resulted in the highest N content in sugar beet (5.60 g/kg) and wheat (4.93 g/kg). The P and K contents followed a similar trend, emphasizing the superior efficacy of integrated fertilization. Overall, the results highlight the superior performance of T4 in improving N, P, and K content in both wheat and sugar beet. Sugar beet consistently exhibited higher nutrient content across treatments compared to wheat, underscoring its relatively better adaptation to salinity and nutrient availability in marginally saline soil (**Figure 5**).

### 3.9 Wheat yield

The T4 resulted in an FM of 90.7 g/plant and a DM of 60.8 g/plant, significantly outperforming T1 (control) which recorded 68 g/plant and 42.1 g/plant, respectively (**Table 3**). This was followed closely by T3 with FM and DM values of 90.8 g/plant and 54.6 g/plant. Treatment T5 also yielded higher FM and DM compared to the control but was less effective than T4 and T3. Plant length and spike length showed similar trends. The T4 achieved the tallest plants (104.3 cm) and the longest spikes (13 cm), surpassing T1 (98.2 cm and 9.4 cm). Treatments T3 and T2 exhibited intermediate improvements, whereas T5 showed moderate results.

**Table 3:** Changes in yield and yield components of wheat (*Triticum aestivum* L., cv. Sakha 93) plants cultivated in marginally saline soil after five different agronomic practices (T1-T5)^¥^.

Parameter	T1	T2	T3	T4	T5
Fresh mass (g/plant)	68 ± 0.12c	65.6 ± 0.15c	90.8 ± 0.16a	90.7 ± 0.16a	78.78 ± 0.09b
Dry mass (g/plant)	42.1 ± 0.71c	44.4 ± 0.77c	54.6 ± 0.95b	60.8 ± 1.1a	50.48 ± 0.44b
Plant length (cm)	98.2 ± 2.54b	101.1 ± 2.62a	102.9 ± 2.67a	104.3 ± 2.70a	101.63 ± 1.32a
Spike length (cm)	9.4 ± 0.25c	11 ± 0.15b	11.7 ± 0.16b	13 ± 0.17a	11.28 ± 0.08b
Number of grain (spike^-1^)	47.7 ± 0.78c	57.4 ± 0.85b	60.8 ± 0.90b	68.7 ± 1.0a	58.65 ± 0.44b
1000-grain weight (g)	57 ± 1.63c	58.7 ± 1.68b	59.7 ± 1.70b	61 ± 1.74a	59.1 ± 0.84b
Grain yield (kg/ha)	3909 ± 96e	4826 ± 107d	5851 ± 342b	6719 ± 541a	5326 ± 611c
CRF^†^	nc^‡^	19.0 ± 0.12d	33.2 ± 0.23b	41.8 ± 0.25a	26.6 ± 0.22c

^¥^ T1: Control (no fertilizers or amendments), ii) T2: Biofertilizer inoculation (*Azotobacter chroococcum* SARS 10 and *Azospirillum lipoferum* SP2) + recommended inorganic P and K, iii) T3: 50% inorganic N and 50% organic manure (sewage sludge and poultry manure in a 1:1 ratio based on N content) + recommended P and K, iv) T4: Biofertilizer + T3, and v) T5: Conventional inorganic NPK fertilizers. The recommended inorganic NPK were calculated per hectare per each crop (wheat and sugar beet) according to the recommendations from the Ministry of Agriculture and Soil Reclamation, Egypt.

^†^ Contribution rate of fertilizers.

^‡^ Not calculated.

Means in the same row followed by different letters are significant according to the Tukey’s test at p ≤ 0.05. Data are means ± SD. (n=10).

Grain yield followed the same pattern, with T4 producing the highest yield at 6719 kg/ha. T3 and T5 also recorded substantial increases (5851 and 5326 kg/ha, respectively), while T2 contributed to a lesser yet significant improvement compared to the control. Additionally, the 1000-grain weight was heaviest in T4 (61 g), followed by T3 (59.7 g), while the control yielded the lightest grains (57 g). The CRF highlighted the efficiency of T2 (41.8%) in utilizing the added nutrients compared to other treatments, underscoring the role of biofertilizers in enhancing nutrient use efficiency under stress conditions.

### 3.10 Sugar beet yield

The T4 recorded the highest root FM at 1368 g/plant and shoot FM at 1107 g/plant, significantly exceeding the control (750 g/plant and 677 g/plant, respectively) (**Table 4**). The T3 followed closely with root and shoot FM values of 1297 g/plant and 1143 g/plant, respectively, while T2 also demonstrated significant improvements over T1. Leaf and root morphology exhibited similar trends. Treatment T4 achieved the longest leaves (78 cm) and the widest root cross-section (12.3 cm), outperforming the control (65.3 cm and 9.1 cm, respectively). The T3 also showed substantial improvements in these parameters. The root-to-shoot ratio was slightly higher in T4 (1.3) compared to other treatments, suggesting better allocation of resources to root development. Root yield, a critical parameter for sugar beet production, was highest under T4 (78.8 tons/ha), with T3 and T5 (recommended N, P, and K) also performing well at 75.4 and 76.2 tons/ha, respectively. T2 demonstrated modest gains compared to T1, indicating the positive impact of biofertilizers alone. The CRF further underscored the efficiency of T4 (30.2%) in enhancing yield compared to other treatments.

**Table 4:** Changes in yield and yield components of sugar beet (*Beta vulgaris* L., cv. Kawemira) plants cultivated in marginally saline soil after five different agronomic practices (T1-T5)^¥^.

Parameter	T1	T2	T3	T4	T5
Root FM (g/plant)	750 ± 23d	1110 ± 32c	1297 ± 33b	1368 ± 35a	1125 ± 25c
Shoot FM (g/plant)	677 ± 20d	1000 ± 30c	1143 ± 32a	1107 ± 29b	1126 ± 27a
Leaf length (cm)	65.3 ± 2.1c	71.0 ± 1.9b	77.3 ± 1.8a	78.0 ± 1.5a	73.3 ± 1.1b
Leaf cross (cm)	14.5 ± 0.2c	18.7 ± 0.3b	19.0 ± 0.2b	20.3 ± 0.2a	19.5 ± 0.4b
Root length (cm)	15.6 ± 0.2d	23.7 ± 0.4b	24.0 ± 0.5b	28.0 ± 0.4a	19.3 ± 0.3c
Root cross (cm)	9.1 ± 0.02c	11.2 ± 0.03b	12.5 ± 0.04a	12.3 ± 0.04a	12.3 ± 0.05a
Root/shoot ratio	1.1 ± 0.03ab	1.2 ± 0.01a	1.2 ± 0.02a	1.3 ± 0.02a	1.2 ± 0.01a
Root yield (ton/ha)	55.0 ± 2.0d	57.4 ± 1.8c	75.4 ± 2.2b	78.8 ± 2.3a	76.2 ± 2.1b
CRF^†^	nc^‡^	4.1 ± 0.08d	27.1 ± 0.08c	30.2 ± 0.09a	27.9 ± 0.07b

^¥^ T1: Control (no fertilizers or amendments), ii) T2: Biofertilizer inoculation (*Azotobacter chroococcum* SARS 10 and *Azospirillum lipoferum* SP2) + recommended inorganic P and K, iii) T3: 50% inorganic N and 50% organic manure (sewage sludge and poultry manure in a 1:1 ratio based on N content) + recommended P and K, iv) T4: Biofertilizer + T3, and v) T5: Conventional inorganic NPK fertilizers. The recommended inorganic NPK were calculated per hectare per each crop (wheat and sugar beet) according to the recommendations from the Ministry of Agriculture and Soil Reclamation, Egypt.

^†^ Contribution rate of fertilizers.

^‡^ Not calculated.

Means in the same row followed by different letters are significant according to the Tukey’s test at p ≤ 0.05. Data are means ± SD. (n=10).

### 3.11 Sugar yield and quality

The highest sugar yield (12.9 ton/ha) was observed in T4, reflecting the combined effect of biofertilizers and co-organic N sources. This treatment outperformed T1 (control, 8.7 ton/ha) by 48.8% (**Table 5**). The T5 produced a sugar yield comparable to T4 (12.9 ton/ha), while T3 closely followed with 12.4 ton/ha. Treatment T2 also improved sugar yield to 9.7 ton/ha, demonstrating the role of biofertilizers in enhancing productivity under salt stress. Sugar content (%) peaked at 17.1% in T2, exceeding the control (15.8%). Treatments T4 and T5 also enhanced sugar content to 16.8% and 16.3%, respectively, while T3 (15.7%) was similar to the control.

**Table 5:** Changes in sugar yield and its quality of sugar beet (*Beta vulgaris* L., cv. Kawemira) plants cultivated in marginally saline soil after five different agronomic practices (T1-T5)^¥^.

Parameter	T1	T2	T3	T4	T5
Sugar (%)	15.8 ± 0.2b	17.1 ± 0.3a	15.7 ± 0.3b	16.8 ± 0.4a	16.3 ± 0.2b
Sugar yield (ton/ha)	8.7 ± 0.2b	9.7 ± 0.3b	12.4 ± 0.3a	12.9 ± 0.4a	12.8 ± 0.2a
Potassium (mmole/100g)	3.07 ± 0.2e	3.44 ± 0.3d	3.96 ± 0.3c	4.89 ± 0.4a	4.19 ± 0.2b
Sodium (mmole/100g)	2.73 ± 0.1a	2.01 ± 0.2c	2.38 ± 0.2b	2.45 ± 0.3b	2.39 ± 0.1b
α –amino (mmole/100g)	2.01 ± 0.1d	2.24 ± 0.2c	2.27 ± 0.2c	2.61 ± 0.3a	2.33 ± 0.1b
Quality (%)	80.5 ± 0.5d	85.5 ± 0.7a	81.7 ± 0.7c	82.8 ± 0.8b	81.2 ± 0.5c
CRF^†^	nc^‡^	10.1 ± 0.1c	29.8 ± 0.2b	32.8 ± 0.3a	32.5 ± 0.3a

^¥^ T1: Control (no fertilizers or amendments), ii) T2: Biofertilizer inoculation (*Azotobacter chroococcum* SARS 10 and *Azospirillum lipoferum* SP2) + recommended inorganic P and K, iii) T3: 50% inorganic N and 50% organic manure (sewage sludge and poultry manure in a 1:1 ratio based on N content) + recommended P and K, iv) T4: Biofertilizer + T3, and v) T5: Conventional inorganic NPK fertilizers. The recommended inorganic NPK were calculated per hectare per each crop (wheat and sugar beet) according to the recommendations from the Ministry of Agriculture and Soil Reclamation, Egypt.

^†^ Contribution rate of fertilizers.

^‡^ Not calculated.

Means in the same row followed by different letters are significant according to the Tukey’s test at p ≤ 0.05. Data are means ± SD. (n=10).

Potassium levels were highest in T4 (4.89 mmol/100 g), indicating enhanced nutrient uptake efficiency, followed by T5 (4.19 mmol/100 g) and T3 (3.96 mmol/100 g). Sodium concentration was lowest in T2 (2.01 mmol/100 g), reflecting its potential in reducing salt stress. Conversely, T4 showed a moderate increase in Na (2.45 mmol/100 g), which may be attributed to higher metabolic activity under improved fertilization. The α-amino nitrogen, an indicator of protein synthesis, was highest in T4 (2.61 mmol/100 g), followed by T5 (2.33 mmol/100 g) and T3 (2.27 mmol/100 g), suggesting that the combined use of biofertilizers and co-organic fertilizers supports N metabolism more effectively. The quality percentage peaked at T2 (85.5%), while T4 and T3 achieved 82.8% and 81.7%, respectively. The CRF was highest in T4 (32.8%) and T5 (32.5%), underscoring their efficiency in improving yield and quality metrics under saline conditions.

### 3.12 Residual effects (soybean bioassay)

**Figure 6:**
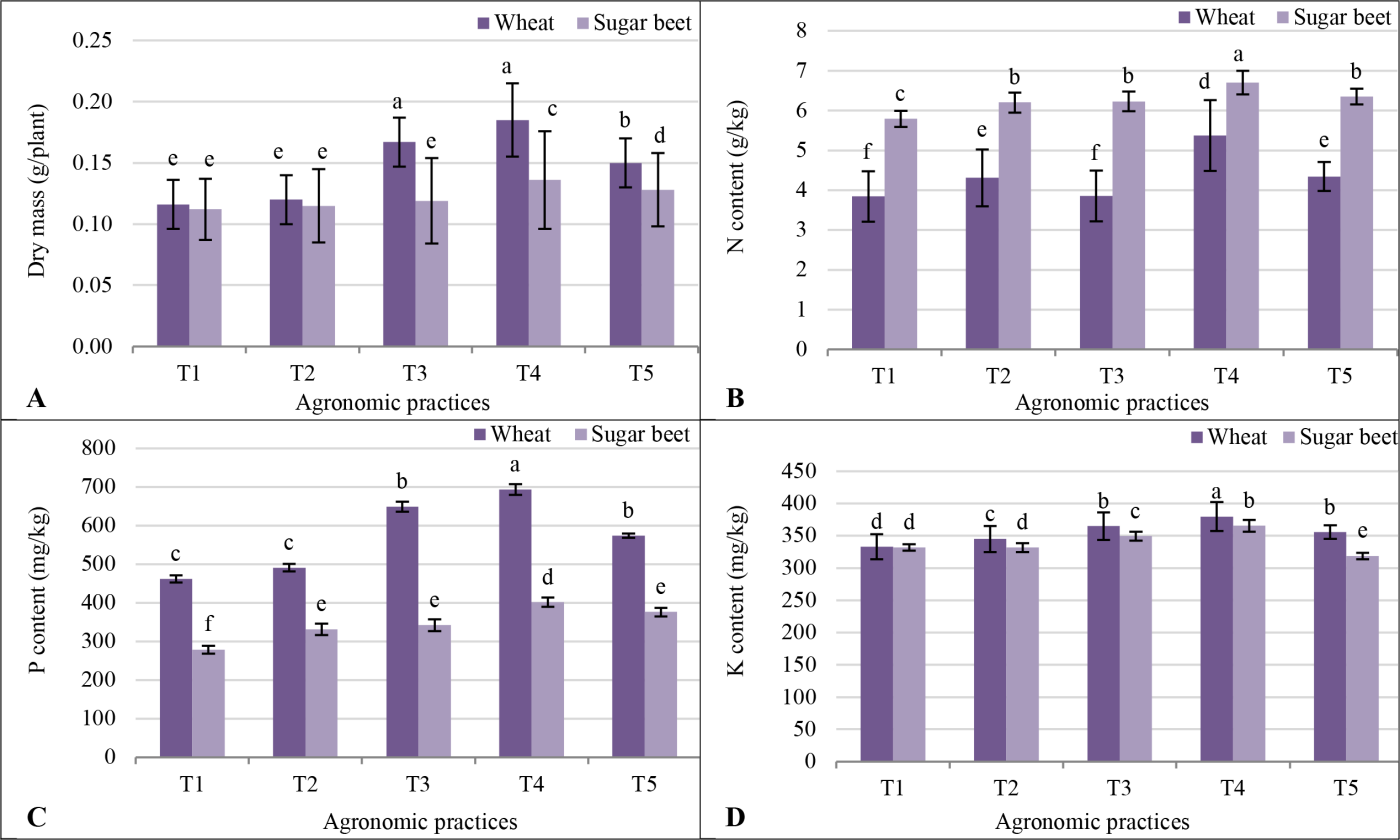
Response of soybean plants grown for 21 days, **(A)** dry mass; **(B)** N content; **(C)** P content; and **(D)** K content, as a model for studying the residual effect of five different agronomic practices (T1-T5) and different vegetative cover (wheat (*Triticum aestivum* L., cv. Sakha 93) and sugar beet (*Beta vulgaris* L., cv. Kawemira)) grown in marginally saline soil. T1: Control (no fertilizers or amendments), ii) T2: Biofertilizer inoculation (*Azotobacter chroococcum* SARS 10 and *Azospirillum lipoferum* SP2) + recommended inorganic P and K, iii) T3: 50% inorganic N and 50% organic manure (sewage sludge and poultry manure in a 1:1 ratio based on N content) + recommended P and K, iv) T4: Biofertilizer + T3, and v) T5: Conventional inorganic NPK fertilizers. The recommended inorganic NPK were calculated per hectare per each crop (wheat and sugar beet) according to the recommendations from the Ministry of Agriculture and Soil Reclamation, Egypt. Different letters on bars are significant according to the Tukey’s test at *p* ≤ 0.05. Data are means ± SD. (n=3).

Treatment T1 resulted in the lowest DM and nutrient content for both crops (**Figure 6**). Treatment T2, with biofertilizers, showed slight growth improvements over T1. However, T4, combining biofertilizers with an organic N source (sewage sludge and poultry manure), achieved the highest DM and nutrient uptake, outperforming all other treatments. The T3 also supported significant growth, though slightly less than T4, emphasizing the value of biofertilizers with organic N sources. The T5 showed moderate growth, surpassing T1 but falling short of T4 and T3. For sugar beet, similar trends emerged. Treatment T1 again yielded the lowest DM and nutrient uptake. The T2 improved growth slightly, while T4 achieved the highest DM and nutrient accumulation, underscoring the synergy of biofertilizers and organic N sources in saline conditions. The T3 performed well but was less effective than T4, and T5 showed intermediate results.

### 3.13 Correlation matrix

**Figure 7:**
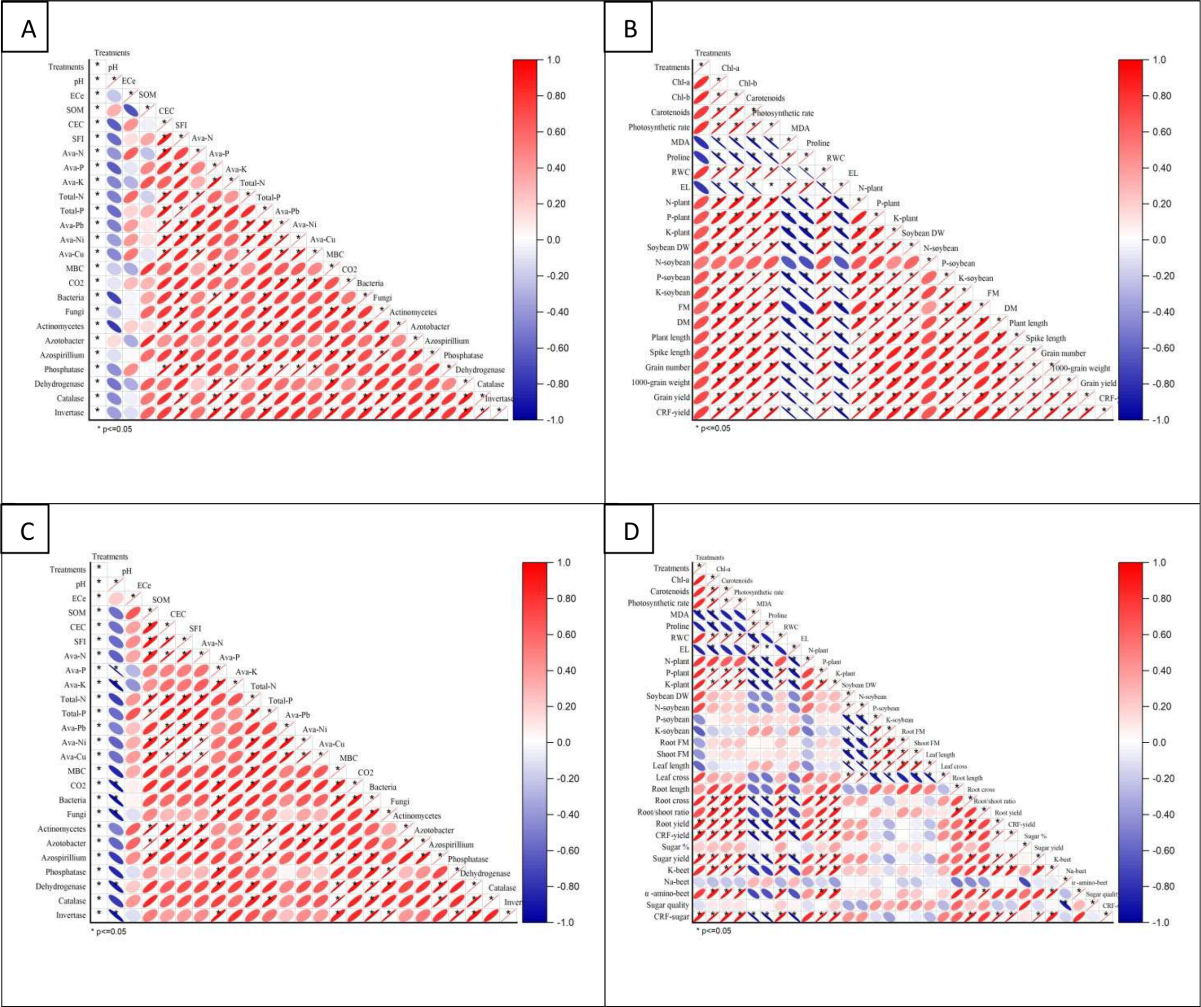
Pearson’s correlation matrix: **(A)** wheat-soil properties, **(B)** wheat-plant properties, **(C)** sugar beet-soil properties, and **(D)** sugar beet-plant properties, of soil and plant parameters of the wheat (*Triticum aestivum* L., cv. Sakha 93) and sugar beet (*Beta vulgaris* L., cv. Kawemira) plants grown in marginally saline soil and treated with the following treatments are: i) T1: Control (no fertilizers or amendments), ii) T2: Biofertilizer inoculation (*Azotobacter chroococcum* SARS 10 and *Azospirillum lipoferum* SP2) + recommended inorganic P and K, iii) T3: 50% inorganic N and 50% organic manure (sewage sludge and poultry manure in a 1:1 ratio based on N content) + recommended P and K, iv) T4: Biofertilizer + T3, and v) T5: Conventional inorganic NPK fertilizers. The recommended inorganic NPK was calculated per hectare for each crop according to the recommendations from the Egyptian Ministry of Agriculture and Land Reclamation.

The correlation matrix for soil and plant parameters after wheat and sugar beet cultivation reveals significant relationships influenced by various treatments (**Figure 7**). Soil pH showed strong positive correlations with EC_e_, SOM, and CEC, indicating higher pH enhances EC_e_, SOM, and CEC. Biofertilizer treatments positively correlated with Total-N and Total-P, suggesting improved nutrient availability. Microbial activity, including bacteria and fungi, positively correlated with Ava-Pb and Ava-Ni, reflecting biofertilizers’ beneficial impact on microbial populations. Enzyme activities like phosphatase and dehydrogenase strongly correlated with Ava-Cu and MBC, indicating better soil health with combined organic and biofertilizer treatments. For plant parameters, Chl a and Chl b exhibited strong positive correlations with photosynthetic rate and carotenoid content, highlighting nutrient treatments’ role in enhancing photosynthetic efficiency. Plant DM, FM (wheat), and DM (sugar beet) positively correlated with N-plant and K-plant, suggesting improved nutrient uptake with biofertilizer and organic amendments. Grain yield and 1000-grain weight showed strong positive correlations with spike length and plant length, with T4 (biofertilizer + organic manure) and T5 yielding the highest values, demonstrating effective integrated nutrient management under saline conditions.

### 3.14 Principal component analysis of soil and plant parameters

**Figure 8:**
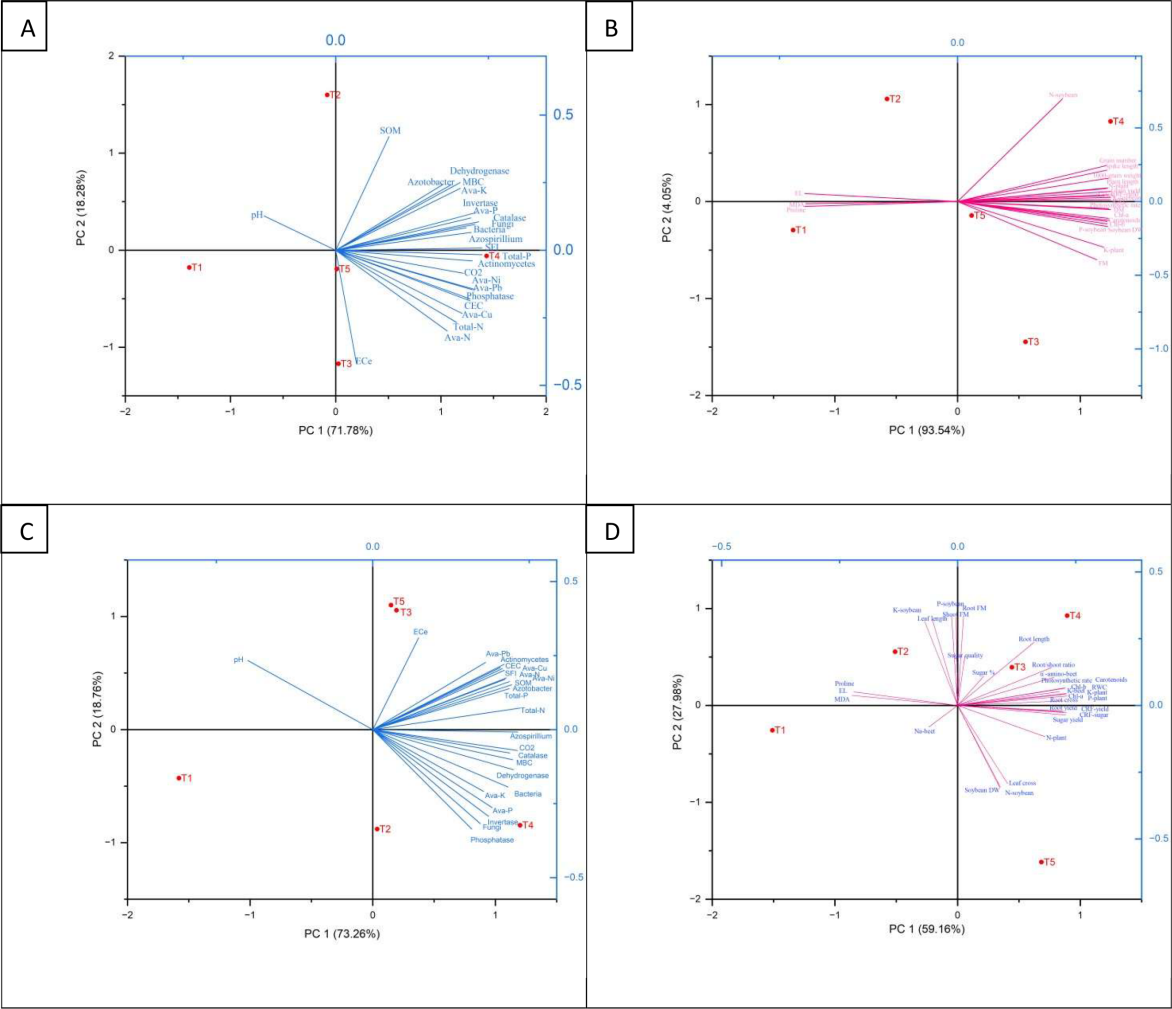
Principal component analysis: **(A)** wheat-soil properties, **(B)** wheat-plant properties, **(C)** sugar beet-soil properties, and **(D)** sugar beet-plant properties, of soil and plant parameters of the wheat (*Triticum aestivum* L., cv. Sakha 93) and sugar beet (*Beta vulgaris* L., cv. Kawemira) plants grown in marginally saline soil and treated with the following treatments are: i) T1: Control (no fertilizers or amendments), ii) T2: Biofertilizer inoculation (*Azotobacter chroococcum* SARS 10 and *Azospirillum lipoferum* SP2) + recommended inorganic P and K, iii) T3: 50% inorganic N and 50% organic manure (sewage sludge and poultry manure in a 1:1 ratio based on N content) + recommended P and K, iv) T4: Biofertilizer + T3, and v) T5: Conventional inorganic NPK fertilizers. The recommended inorganic NPK was calculated per hectare for each crop according to the recommendations from the Egyptian Ministry of Agriculture and Land Reclamation.

The Principal Component Analysis (PCA) for wheat and sugar beet field experiments reveals distinct patterns in soil and plant parameters across treatments (**Figure 8**). For wheat, PC1 (93.54%) and PC2 (4.05%) explain variability. The T1 and T2 cluster near the origin, showing minimal deviation, while T3 aligns negatively with PC1, linked to stress indicators like MDA and proline. Treatments T4 and T5 correlate positively with PC2, associated with enhanced plant parameters such as grain number, 1000-grain weight, spike length, plant length, P-soybean, N-soybean, and soybean DM, indicating improved growth and yield. Soil parameters like EC_e_ and pH negatively correlate with PC1, reflecting stress in T3, while SOM, dehydrogenase, *Azotobacter*, and enzyme activities (phosphatase, catalase, invertase) positively correlate with PC2, highlighting T4 and T5’s benefits on soil health. For sugar beet, PC1 (59.16%) and PC2 (27.98%) account for variability. The T1 is near the origin, T2 shows slight PC1 improvement, while T3 and T5 align negatively with PC1, linked to Na-beet stress. T4 positively correlates with PC2, tied to root length, shoot FM, sugar %, and yield. Soil EC_e_ and pH negatively correlate with PC1, while SOM and enzyme activities align with PC2, emphasizing T4’s positive impact.

## 4 Discussion

### 4.1 Soil health and nutrient availability

The study revealed that the integrated treatment (T4) significantly improved SOM by 23.3 g/kg in wheat and 22.9 g/kg in sugar beet, while reducing EC_e_ to 3.90 dS/m (wheat) and 3.13 dS/m (sugar beet) compared to the control (T1). This aligns with previous studies showing that organic amendments enhance SOM and soil structure, particularly in saline soils (Alshaal et al., 2025; Elhawat et al., 2023; Zuo et al., 2019). The reduction in EC_e_ following the application of poultry manure and sewage sludge can be explained by several complementary physicochemical and biological mechanisms reported in saline and salt-affected soils. First, both poultry manure and sewage sludge are rich in organic matter, which increases soil CEC. The higher CEC enhances the adsorption of soluble salts, particularly Na^+^, onto organic and mineral exchange sites, thereby decreasing the concentration of salts in the soil solution and reducing EC_e_ (Alavilli et al., 2023). Second, these organic amendments supply appreciable amounts of divalent cations such as Ca^2+^ and Mg^2+^, which displace Na^+^ from soil exchange complexes through cation exchange reactions. The displaced Na^+^ becomes more mobile and can be leached below the root zone under irrigation or rainfall, resulting in a net decline in soil EC_e_ (García-Berumen et al., 2025). Third, the incorporation of organic amendments improves soil physical properties, including aggregate stability, porosity, and hydraulic conductivity. Improved soil structure enhances water infiltration and percolation, facilitating the downward movement and leaching of soluble salts and preventing their accumulation in the surface soil layers (Luo et al., 2018).

In addition, microbial decomposition of poultry manure and sewage sludge stimulates the production of low-molecular-weight organic acids and CO_2_, which promote soil aggregation and reduce salt concentration in the rhizosphere by improving soil permeability and ion redistribution (Luo et al., 2018). When combined with biofertilizers, these effects are further amplified through enhanced root growth, rhizosphere acidification, and microbial activity, which collectively improve soil structure and promote more efficient salt leaching (Alshaal et al., 2025), explaining the greater EC_e_ reduction observed under integrated treatments.

The reduction in EC_e_ under T4 suggests that organic amendments and biofertilizers facilitate salt leaching and improve soil physical properties. These findings are consistent with research by Luo et al. (2018), who reported that organic amendments enhance soil water retention and reduce salinity stress. This dual amelioration stems from organic matter-induced cation displacement and microbial acidification. Sewage sludge and poultry manure contributed high CEC organic colloids, which adsorbed Na^+^, lowering EC_e_ via leaching facilitation during irrigation (Alavilli et al., 2023).

Concurrently, *Azotobacter* and *Azospirillum* stimulated heterotrophic respiration, releasing organic acids (e.g., gluconic, citric) that protonated exchange sites, reducing pH and enhancing P solubility (Vincze et al., 2024). Unlike T5, T4 sustained pH moderation through continuous microbial C turnover, as evidenced by 58% higher substrate-induced respiration in wheat.

The integrated treatment (T4) significantly improved soil CEC; this aligns with previous studies showing that organic amendments enhance CEC by increasing SOM and improving nutrient retention (Alshaal et al., 2025; Luo et al., 2018; Zuo et al., 2019). The integrated treatment (T4) significantly improved soil CEC through the addition of high-CEC organic colloids from sewage sludge and poultry manure, which increased surface area and negative charge density via humified organic matter formation, thereby enhancing nutrient retention and buffering against salinity-induced Na^+^ saturation (Zuo et al., 2019). This was complemented by biofertilizer-induced root proliferation and exopolysaccharide production by *Azotobacter chroococcum* and *Azospirillum lipoferum*, which stabilized soil aggregates and preserved exchange sites (Vincze et al., 2024).

The SFI also improved under T4, indicating an enhancement in soil health. The resulting SFI improvement under T4 reflected synergistic gains in multiple parameters, e.g., SOM, CEC, and nutrient pools, driven by enhanced microbial biomass that accelerated organic matter mineralization and aggregate stability (Qi et al., 2009). Nutrient availability (Ava-N and Ava-K) was also significantly improved under T4, which can be attributed to the role of biofertilizers in nitrogen fixation and nutrient mobilization, as well as the gradual release of nutrients from organic amendments (Alshaal et al., 2024; Hayat et al., 2010). Elevated Ava-N and Ava-K in wheat under T4 arose from biological N_2_ fixation (up to 25–30 kg N/ha by *Azotobacter*–*Azospirillum* consortia) and K^+^ solubilization via organic acid excretion (e.g., gluconic, citric acids) that displaced exchangeable K^+^ from clay interlayers (Alshaal et al., 2024; Hayat et al., 2010; Zhang et al., 2025). The gradual nutrient release from organic amendments prevented leaching and synchronized supply with crop demand, unlike the pulse release in T5 (Hoover et al., 2019). The increase in Ava-P under T4 further supports the synergistic effects of biofertilizers and organic amendments in enhancing nutrient availability (Kumar et al., 2022a). Moreover, Ava-P may be increased due to phosphatase enzyme induction by PGPR and pH-dependent P desorption from Fe/Al oxides under mild acidification, facilitated by organic anion complexation (Kumar et al., 2022b). Total-N and Total-P were highest under T4; this is consistent with research by Luo et al. (2018), who reported that organic amendments and biofertilizers improve nutrient availability in saline soils. Total-N and Total-P accumulation in T4 resulted from reduced N volatilization (via microbial immobilization) and P fixation (via Ca-organic complexes), creating a sustained nutrient reservoir (Luo et al., 2018). The availability of heavy metals (Ava-Cu, Ava-Ni, and Ava-Pb) was also higher under T4, suggesting that organic amendments enhance metal mobilization. However, the levels remained within safe limits, supporting the safe use of sewage sludge in agriculture (Tsadilas et al., 1995). The higher availability of DTPA-extractable Cu, Ni, and Pb under T4 was mediated by chelating ligands (e.g., fulvic acids, amino acids) from decomposing organic matter and microbial siderophores, which mobilized metals without exceeding phytotoxic thresholds, confirming the safe agronomic use of sewage sludge when co-applied with biofertilizers that enhance metal–organic binding and reduce bioavailability (Achkir et al., 2023; Tsadilas et al., 1995).

### 4.2 Soil biochemical and microbiological features

The MBC increased significantly under the T4 treatment; this aligns with recent studies, indicating that organic amendments enhance microbial activity (Shu et al., 2022). The increase in MBC under the T4 treatment may be due to the synergistic provision of labile carbon substrates from sewage sludge and poultry manure, which fueled copiotrophic microbial proliferation, while *Azotobacter chroococcum* and *Azospirillum lipoferum* secreted exopolysaccharides and siderophores that protected cells from salinity-induced osmotic stress, thereby enhancing biomass accumulation (Shu et al., 2022; Vincze et al., 2024).

The CO_2_ emissions were also highest under T4, reflecting increased microbial respiration and organic matter decomposition (Cui et al., 2023). The highest CO_2_ emissions under T4 reflected accelerated heterotrophic respiration driven by enhanced organic matter decomposition, as the high C/N ratio substrates stimulated fast-growing r-strategists (e.g., *Pseudomonas*, *Bacillus*), with biofertilizer-induced root exudates (simple sugars, amino acids) further priming microbial catabolism and C turnover (Cui et al., 2023; Mahmud et al., 2021).

The total count of bacteria, fungi, actinomycetes, *Azotobacter*, and *Azospirillum* increased significantly under T4, with *Azotobacter* counts, and these findings are consistent with research by El-Ramady et al. (2018) and Kumar et al. (2022b), who demonstrated that PGPR enhance microbial populations and nutrient cycling in soil. The elevated total counts of bacteria, fungi, actinomycetes, *Azotobacter*, and *Azospirillum* under T4 resulted from niche diversification: organic amendments created microhabitats rich in organic C and moisture, while PGPR inoculation provided competitive colonizers that fixed N_2_ (up to 30 kg/ha) and produced IAA and ACC deaminase, promoting rhizosphere competence and suppressing salt-sensitive taxa (El-Ramady et al., 2018; Kumar et al., 2022b). Soil enzymes play a critical role in nutrient cycling and organic matter decomposition, and their activity is closely linked to soil health and fertility. Phosphatase enzymes are essential for P mineralization, converting organic P into plant-available forms. The increase in phosphatase activity under T4 can be attributed to the combined effects of biofertilizers and organic amendments. Biofertilizers, particularly *Azotobacter* and *Azospirillum*, produce organic acids that solubilize P, while organic amendments provide a substrate for microbial activity, enhancing enzyme production (Meena et al., 2020). This aligns with findings by Shahwar et al. (2023), who reported that biofertilizers increase phosphatase activity by stimulating microbial populations. Phosphatase activity surged under T4 through dual P-solubilization mechanisms: *Azotobacter* and *Azospirillum* excreted organic acids (gluconic, citric) that lowered rhizosphere pH and chelated Ca^2+^, releasing bound P, while organic matter decomposition supplied phosphatase-inducing substrates (e.g., phospholipids, nucleic acids), upregulating microbial alkaline and acid phosphatase gene expression (Meena et al., 2020; Shahwar et al., 2023).

Dehydrogenase is an indicator of microbial metabolic activity and soil health. The increase in dehydrogenase activity under T4 reflects enhanced microbial activity due to the addition of organic amendments and biofertilizers. Organic amendments provide C and energy sources for microorganisms, while biofertilizers promote microbial growth and activity (De Andrade et al., 2023). This is consistent with study of Al Tawaha et al. (2025), who demonstrated that biofertilizers enhance dehydrogenase activity by increasing microbial biomass and activity. Dehydrogenase activity increased due to elevated intracellular oxidoreductase function in an expanded microbial community, where poultry manure’s labile C served as an electron donor and biofertilizers enhanced membrane integrity via osmoprotectants, boosting overall metabolic intensity under salinity (Al Tawaha et al., 2025; De Andrade et al., 2023).

Catalase enzymes mitigate oxidative stress by breaking down hydrogen peroxide (H_2_O_2_) into water and oxygen. The increase in catalase activity under T4 suggests reduced oxidative stress in the soil. Organic amendments improve soil structure and reduce salinity stress, while biofertilizers enhance microbial activity, leading to increased enzyme production (Luo et al., 2018). This is supported by research by Shi et al. (2019), who reported that organic amendments reduce oxidative stress by improving soil health. Catalase activity rose under T4 as a stress-responsive adaptation: organic amendments improved soil aeration and aggregate stability, reducing H_2_O_2_ accumulation from salinity-induced ROS, while PGPR-triggered antioxidant enzyme synthesis in microbes (e.g., katA gene upregulation) decomposed peroxides, mitigating oxidative damage to cellular components (Luo et al., 2018; Shi et al., 2019).

Invertase enzymes are involved in carbohydrate metabolism, breaking down sucrose into glucose and fructose. The increase in invertase activity under T4 reflects enhanced microbial activity and organic matter decomposition. Biofertilizers and organic amendments provide substrates for microbial growth, increasing enzyme production (Meena et al., 2020). This aligns with findings by Kumar et al. (2022a), who demonstrated that biofertilizers enhance invertase activity by promoting microbial populations. Invertase activity was enhanced by sucrose-rich root exudates (especially in sugar beet) and fungal/actinomycete enrichment under T4, where organic substrates induced sucrase gene expression (invA), accelerating sucrose hydrolysis into glucose and fructose to support microbial energy demands and osmotic adjustment in saline conditions (Kumar et al., 2022a; Meena et al., 2020).

Although soil respiration rate and dehydrogenase activity were higher under wheat (up to 52% and 41% greater than sugar beet under T4, respectively), invertase activity was consistently higher under sugar beet (up to 38% greater than wheat under T4). This inverse pattern does not violate direct proportionality among enzyme activities but reflects crop-specific rhizosphere dynamics and substrate-driven microbial selection. Wheat, a C_3_ cereal with extensive fibrous roots, exudes simple sugars and amino acids that stimulate fast-growing copiotrophic bacteria (e.g., *Pseudomonas*, *Bacillus*), driving elevated respiration and dehydrogenase activity (indicator of general oxidoreductase function) (Nannipieri et al., 2018). Under saline conditions, increased root turnover in wheat releases more labile carbon, thereby further enhancing overall microbial metabolism (Mahmud et al., 2021; Zhang et al., 2026a). In contrast, sugar beet, a C_4_ root crop, allocates more photosynthates to sucrose-rich root exudates, selectively enriching fungal and actinomycete populations that produce invertase for sucrose hydrolysis (Hoffmann, 2010). The higher invertase in sugar beet rhizosphere aligns with its greater demand for C mobilization during root bulking, even under salinity (Raffi and Charyulu, 2021). Thus, respiration/dehydrogenase reflects total microbial energy flux (favored in wheat), while invertase reflects sucrose-specific C cycling (favored in sugar beet). These complementary patterns reinforce the efficacy of T4 in stimulating functionally diverse microbial guilds across crop systems.

### 4.3 Plant stress indicators

Salinity stress triggers oxidative damage and osmotic stress in plants, resulting in diminished growth and productivity. The integrated treatment (T4) significantly alleviated stress indicators in both wheat and sugar beet. MDA, a marker of lipid peroxidation and oxidative damage, was reduced under T4, indicating lower oxidative stress. Biofertilizers enhance antioxidant enzyme activity, while organic amendments improve soil structure and mitigate salinity stress (Abd Allah et al., 2015). These findings are consistent with research by Luo et al. (2018), who demonstrated that organic amendments reduce oxidative stress by enhancing soil health. The reduced MDA content under T4 resulted from enhanced antioxidant enzyme activity triggered by *Azotobacter* and *Azospirillum*-derived ACC deaminase and IAA, which lowered ethylene levels and upregulated ROS-scavenging pathways, while organic amendments supplied phenolic compounds and humic substances that directly quenched free radicals, thereby minimizing lipid peroxidation in cell membranes (Abd Allah et al., 2015; Luo et al., 2018).

Proline, an osmoprotectant that accumulates under stress to maintain cellular osmotic balance, was also reduced under T4, suggesting improved osmotic balance and reduced stress. Biofertilizers enhance nutrient uptake and water retention, while organic amendments improve soil structure, reducing the need for proline accumulation (Etesami and Beattie, 2018). This aligns with studies by Munns and Gilliham (2015), who showed that biofertilizers alleviate osmotic stress in plants. The decrease in proline accumulation under T4 reflected improved cellular water potential and reduced osmotic stress, as biofertilizers promoted K^+^ uptake via HKT1 and AKT1 transporter induction, maintaining turgor pressure and reducing the need for proline synthesis, while organic matter-enhanced soil aggregation increased water-holding capacity and stabilized rhizosphere EC_e_, preventing cellular dehydration (Etesami and Beattie, 2018; Munns and Gilliham, 2015).

The RWC, an indicator of plant water status, increased under T4, reflecting enhanced water retention and reduced salinity stress. Organic amendments improve soil water-holding capacity, while biofertilizers promote root growth and water uptake (Razaq et al., 2017). This is supported by research by Alharbi et al. (2022a, b), who reported that biofertilizers enhance water status in marginally saline soil. The RWC increased under T4 due to extensive root system development stimulated by PGPR-produced gibberellins and cytokinins, which enhanced root hair density and hydraulic conductivity, coupled with improved soil porosity from organic amendments that reduced bulk density and facilitated capillary water movement, ensuring sustained xylem flux under saline conditions (Alharbi et al., 2022b; Razaq et al., 2017).

The EL, a measure of membrane stability, was reduced under T4, indicating improved membrane integrity and reduced stress. Biofertilizers enhance antioxidant activity, protecting cell membranes from oxidative damage, while organic amendments mitigate salinity stress (Abd Allah et al., 2015). These findings are consistent with Mącik et al. (2020), who demonstrated that biofertilizers improve membrane stability under stress conditions. The lower EL under T4 indicated preserved membrane integrity, achieved through biofertilizer-mediated phospholipid reinforcement via osmolytes (e.g., glycine betaine) and organic amendment-derived Ca^2+^ bridging of membrane lipids, which stabilized phospholipid bilayers against Na^+^-induced displacement, while upregulated aquaporin expression (PIP1, PIP2) maintained selective ion permeability, collectively shielding cellular compartments from salinity damage (Abd Allah et al., 2015; Mącik et al., 2020).

### 4.4 Efficiency of photosynthetic machinery

Photosynthesis is a critical process for plant growth and productivity, and its efficiency is often impaired under salinity stress (Mahmud et al., 2021). Treatment T4 significantly improved photosynthetic efficiency in both wheat and sugar beet. Chlorophyll a and b are essential pigments for capturing light energy during photosynthesis. The increase in chlorophyll a and chlorophyll b under T4 can be attributed to improved nutrient availability and reduced salinity stress. Biofertilizers enhance N_2_ fixation and nutrient uptake, while organic amendments improve soil structure and water retention, reducing osmotic stress (Kumar et al., 2022a). This is consistent with findings by Alharbi et al. (2022a), who reported that biofertilizers improve chlorophyll content by enhancing nutrient availability. Carotenoids protect chlorophyll from oxidative damage and play a role in light harvesting. The increase in carotenoid content under T4 reflects reduced oxidative stress and improved photosynthetic efficiency. Organic amendments and biofertilizers enhance antioxidant activity, protecting photosynthetic pigments from damage (Ali et al., 2018). This aligns with studies by Munns and Tester (2008), who demonstrated that biofertilizers reduce oxidative stress and improve carotenoid content. The photosynthetic rate peaked under T4, reflecting improved light capture and carbon fixation. Biofertilizers enhance nutrient uptake, particularly N and P, which are essential for photosynthesis, while organic amendments improve water retention and reduce salinity stress (Tian-gen et al., 2017). This is supported by research by Khayamim (2022), who reported that integrated nutrient management enhances photosynthetic efficiency in sugar beet.

The T4 treatment significantly enhanced photosynthetic efficiency in wheat and sugar beet under salinity by alleviating nutrient limitations, reducing oxidative stress, and improving C assimilation pathways. The increase in chlorophyll a and b under T4 resulted from enhanced N availability via *Azotobacter*-mediated N_2_ fixation and *Azospirillum*-induced ammonium (NH_4_^+^) assimilation, which upregulated glutamine synthetase and chlorophyll biosynthesis genes, while organic amendments supplied ferrous (Fe^2+^) and magnesium (Mg^2+^) ions through chelation, preventing chlorosis and stabilizing photosystem II reaction centers (Alharbi et al., 2022b; Kumar et al., 2022b). Carotenoid content rose under T4 due to PGPR-triggered upregulation of carotenoid biosynthesis via IAA signaling, which enhanced xanthophyll cycle activity to dissipate excess light energy and quench singlet oxygen, while humic substances from organic matter acted as direct antioxidants, protecting D1 protein from salinity-induced photoinhibition (Ali et al., 2018; Munns and Gilliham, 2015). The peak photosynthetic rate under T4 was driven by improved stomatal conductance and RuBisCO activase activity, facilitated by biofertilizer-enhanced K^+^ uptake that maintained guard cell turgor, and organic amendment-induced soil water retention that sustained CO_2_ diffusion, while P availability supported ATP synthesis in the Calvin cycle, collectively overcoming salinity-limited electron transport (Khayamim, 2022; Tian-gen et al., 2017).

### 4.5 NPK building up in plant tissues

The increase in N content under T4 can be attributed to the N_2_-fixing capabilities of *Azotobacter* and *Azospirillum*. These biofertilizers fix atmospheric N_2_ and convert it into plant-available forms, enhancing N uptake (Aasfar et al., 2024). Additionally, organic amendments like poultry manure and sewage sludge provide a slow-release source of N, ensuring sustained nutrient availability (Elmeknassi et al., 2024). This aligns with findings by Jin et al. (2022), who reported that organic amendments improve N availability and uptake in wheat. The increase in P content under T4 is linked to the P-solubilizing activity of biofertilizers. *Azotobacter* and *Azospirillum* produce organic acids that solubilize insoluble P compounds, making P more available to plants (Kumar et al., 2022a). Organic amendments also enhance P availability by improving soil microbial activity and SOM (Luo et al., 2018). This is consistent with research by Alavilli et al. (2023), who demonstrated that biofertilizers and organic amendments enhance P uptake in sugar beet. The increase in K content under T4 reflects improved K availability and uptake. Biofertilizers enhance root growth and nutrient absorption, while organic amendments provide a source of K and improve soil structure, facilitating K uptake (Zuo et al., 2019). This is supported by studies by Arshad et al. (2024), who reported that organic amendments improve K availability and uptake in wheat.

### 4.6 Yield and yield components of wheat and sugar beet

The integrated treatment (T4) significantly improved yield and yield components in both wheat and sugar beet. Wheat grain yield reached 6719 kg/ha and sugar beet root yield 78.8 tons/ha under T4, outperforming the control by substantial margins and conventional NPK. Wheat grain yield was highest under T4, with significant improvements in FM, DM, plant height, spike length, and 1000-grain weight. The increase in yield can be attributed to improved nutrient availability, enhanced photosynthetic efficiency, and reduced stress. Biofertilizers enhance root growth and nutrient uptake, while organic amendments improve soil structure and water retention, supporting higher yields (Alharbi et al., 2022a). This aligns with findings by Jin et al. (2022), who reported that organic amendments and biofertilizers enhance wheat yield under saline conditions. Sugar beet root yield also peaked under T4, with improvements in root and shoot FM, leaf length, and root width. The increase in yield reflects improved nutrient availability, water retention, and stress tolerance. Biofertilizers enhance root growth and nutrient uptake, while organic amendments improve soil structure and organic matter content, supporting higher yields (Khayamim, 2022). This is consistent with research by Alavilli et al. (2023), who demonstrated that integrated nutrient management enhances sugar beet yield under saline conditions.

### 4.7 Sugar yield and its quality

The integrated treatment (T4) significantly improved sugar yield and quality in sugar beet. Sugar yield was highest under T4, reflecting improved nutrient availability and photosynthetic efficiency. Biofertilizers enhance nutrient uptake and root growth, while organic amendments improve soil structure and water retention, supporting higher sugar yields (Khayamim, 2022). This aligns with findings by Luo et al. (2018), who reported that organic amendments enhance sugar yield in sugar beet. Sugar content peaked at 16.8% under T4, with improvements in K content and reductions in Na content. The increase in sugar quality reflects improved nutrient uptake and stress tolerance. Biofertilizers enhance nutrient availability and root growth, while organic amendments improve soil structure and water retention, supporting higher sugar quality (Alavilli et al., 2023).

### 4.8 Safe production of wheat and sugar beet

The availability of heavy metals (Ava-Cu, Ava-Ni, and Ava-Pb) in soil remained within safe limits under all treatments, supporting the safe use of sewage sludge in agriculture. Organic amendments improve soil structure and reduce heavy metal toxicity, while biofertilizers enhance microbial activity, reducing metal availability (Tsadilas et al., 1995). This is supported by studies by Elmeknassi et al. (2024), who reported that organic amendments do not significantly increase heavy metal toxicity in crops. The safe production of wheat and sugar beet under T4 reflects the combined effects of biofertilizers and organic amendments in reducing heavy metal toxicity and improving soil health. Biofertilizers enhance microbial activity and nutrient uptake, while organic amendments improve soil structure and water retention, supporting safe production (Zuo et al., 2019). This aligns with findings by Luo et al. (2018), who reported that organic amendments enhance safe crop production in saline soils.

The availability of heavy metals in the soil remained within safe limits under all treatments. This was especially evident under T4. The reduced availability was mainly due to immobilization processes associated with the high organic matter content in sewage sludge and poultry manure. These materials enhanced several mechanisms. They promoted adsorption onto mineral surfaces and complexation with organic ligands such as fulvic and humic acids. They also supported surface precipitation and ion-exchange reaction (Shu et al., 2022; Tsadilas et al., 1995). This was further supported by biofertilizer-induced microbial activity, where *Azotobacter chroococcum* and *Azospirillum lipoferum* stimulated heterotrophic bacteria and fungi that secreted chelators (e.g., siderophores) and exopolysaccharides, facilitating metal sequestration and redox transformations (e.g., reduction of more toxic forms), which collectively minimized toxicity without exceeding phytotoxic thresholds (Elmeknassi et al., 2024; El-Ramady et al., 2018). The safe production of wheat and sugar beet under T4 reflected these synergistic effects, as organic amendments improved soil aggregation and water retention, buffering salinity and reducing metal-induced osmotic stress, while biofertilizers enhanced rhizosphere nutrient uptake (e.g., K^+^ competition with metals) and antioxidant enzyme activity in plants, promoting root proliferation and biomass accumulation without heavy metals bioaccumulation in edible tissues (Luo et al., 2018; Zuo et al., 2019).

### 4.9 Residual effects (soybean bioassay)

The bioassay trial using soybean demonstrated that the integrated treatment (T4) significantly improved soil quality. This was evidenced by elevated FM, DM, and NPK content in soybean plants grown in soil from T4. The increase in N content in soybean under T4 can be attributed to the N_2_-fixing capabilities of *Azotobacter* and *Azospirillum*. These biofertilizers fix atmospheric N_2_ and convert it into plant-available forms, enhancing N availability in the soil (Aasfar et al., 2024). Additionally, organic amendments like poultry manure and sewage sludge provide a slow-release source of N, ensuring sustained nutrient availability (Elmeknassi et al., 2024). This aligns with findings by Jin et al. (2022), who reported that organic amendments improve N availability and uptake in crops. The increase in P content in soybean under T4 is linked to the phosphorus-solubilizing activity of biofertilizers. *Azotobacter* and *Azospirillum* produce organic acids that solubilize insoluble P compounds, making P more available to plants (Kumar et al., 2022a). Organic amendments also enhance P availability by improving soil microbial activity and organic matter content (Luo et al., 2018). This is consistent with research by Alavilli et al. (2023), who demonstrated that biofertilizers and organic amendments enhance P uptake in crops. The increase in K content in soybean under T4 reflects improved K availability and uptake. Biofertilizers enhance root growth and nutrient absorption, while organic amendments provide a source of K and improve soil structure, facilitating K uptake (Zuo et al., 2019).

### 4.10 Salinity challenges and scalability of the integrative approach

Soil salinity poses a multifaceted challenge to global agriculture, affecting over 1 billion hectares and causing annual economic losses exceeding $27 billion (Singh, 2022). It induces osmotic stress that limits water uptake, ionic toxicity from Na^+^ and Cl^-^ accumulation that disrupts enzymatic function and K^+^/Na^+^ homeostasis, nutrient imbalances that impair uptake of essential elements like P and K, and secondary sodicity that degrades soil structure through clay dispersion (Alsaeedi et al., 2017). Wheat, highly sensitive to salinity, experiences yield declines of approximately 7.1% per dS/m above a threshold of 6 dS/m, with severe impacts during germination and tillering due to reduced cell turgor and premature senescence (Rehman et al., 2025). Sugar beet, while moderately tolerant with a threshold near 7 dS/m, suffers root growth inhibition, reduced sucrose accumulation, and biomass loss beyond 10 dS/m, compromising both yield and quality (Wang et al., 2024). The experimental soil, starting at EC_e_ = 3.47 dS/m, represents moderate salinity a range where organic amendments and microbial interventions exert maximum remedial effects through organic acid production, Na^+^ chelation, and improved hydraulic conductivity. The T4 treatment reduced EC_e_ by 11–20% via enhanced cation exchange, K^+^/Ca^2+^ displacement of Na^+^ from manure-derived inputs, and increased soil aggregation that promoted salt leaching during irrigation.

The scalability of this integrative fertilization strategy extends effectively across salinity levels of 2–8 dS/m and soil textures including silty loam, clay loam, and loamy sand, where CEC exceeds 20 cmolc/kg to buffer Na^+^ accumulation. The salt-tolerant PGPR strains maintain N_2_ fixation and phytohormone synthesis up to 8–10 dS/m, while the balanced C/N ratio (~6.7) from blended organic wastes sustains microbial activity and gradual nutrient release, countering salinity-suppressed mineralization. In arid and semi-arid areas with regulated irrigation—such as the Nile Delta, Mediterranean regions, and salt-affected zones of the Middle East—this strategy fits well with locally available waste streams and can lower inorganic fertilizer costs by about 42%, resulting in net returns of approximately $380–450 per hectare (El-Ramady et al., 2019). The T4 strategy offers practical lessons for similar marginally saline areas (EC_e_ 2–8 dS/m) common in the Nile Delta, Mediterranean basins, and other arid/semi-arid regions. By substituting 50% inorganic N with locally available blended wastes and salt-tolerant PGPR, farmers can reduce fertilizer costs by approximately 42%, improve soil health persistently (as shown in the soybean bioassay), and maintain or increase yields without heavy metal risks. This integrative approach promotes circular economy principles by valorizing urban and animal wastes, enhances resilience to salinity through combined physicochemical and biological mechanisms, and supports sustainable intensification in resource-constrained smallholder systems.

## 5 Conclusion

The present study demonstrated that the integrated application of biofertilizers (*Azotobacter chroococcum* and *Azospirillum lipoferum*) with combined organic N sources (sewage sludge and poultry manure; T4) was the most effective treatment for improving soil quality and crop performance in moderately saline soil (EC_e_ ≈ 3.5 dS/m). This treatment significantly reduced soil salinity (EC_e_), increased SOM, and enhanced microbial biomass, enzyme activities (dehydrogenase, phosphatase, catalase, and invertase), and beneficial microbial populations, indicating improved soil biochemical and biological functioning. At the plant level, T4 markedly enhanced nutrient acquisition (N, P, and K) in both wheat and sugar beet, improved photosynthetic pigments and photosynthetic rate, and alleviated salinity-induced stress, as evidenced by lower malondialdehyde and proline contents, reduced electrolyte leakage, and higher relative water content. These physiological improvements translated into significant increases in wheat grain yield and yield components, as well as sugar beet root yield, sugar yield, and sugar quality, with reduced Na accumulation and improved K^+^/Na^+^ balance. Results of PCA further confirmed the strong positive association between T4, improved soil health indicators, and superior plant growth and yield attributes. The residual bioassay using soybean confirmed that soils receiving T4 maintained higher fertility, reflected by increased biomass production and N:P:K uptake, highlighting the persistence of treatment benefits beyond the primary crops. Importantly, the availability of Cu, Ni, and Pb remained within safe limits under all treatments, supporting the agronomic safety of using sewage sludge when combined with biofertilizers and organic amendments. Overall, the results indicate that integrating biofertilizers with organic N sources can effectively improve soil functionality, mitigate salinity stress, and enhance the productivity and quality of wheat–sugar beet cropping systems under moderate salinity. While these findings demonstrate clear agronomic and environmental benefits under the tested conditions, longer-term field studies across different salinity levels, soil textures, and climatic zones are required to validate the long-term sustainability and broader applicability of this integrated fertilization strategy.

## Data Availability

The original contributions presented in the study are included in the article/**Supplementary Material**. Further inquiries can be directed to the corresponding author.
