# Supplementary material for: Synergistic effects of nitrogen, sewage sludge, and poultry manure on integrative fertilization for wheat and sugar beet in marginally saline soil
Source: Front Plant Sci. 2026 May 22;17:1827328. doi: 10.3389/fpls.2026.1827328 (PMC13236655; doi:10.3389/fpls.2026.1827328)
Supplement: Supplementary file 1 [file Table1.docx]

| **Table S1:** Physical, chemical, and microbiological traits of experimental soil, sewage sludge, and poultry manure. | | | |
| --- | --- | --- | --- |
| **Parameter** | **Soil** | **Sewage sludge** | **Poultry manure** |
| pH^*^ | 7.88±0.01 | 5.54±0.02 | 7.30±0.01 |
| Electrical conductivity (EC_e_; soil paste extract at 25ºC; dS/m) | 3.47±0.05 | 7.27±0.08 | 9.24±0.08 |
| Saturation percentage (%) | 77±1 | 164±2 | 125±2 |
| Total organic carbon (TOC; %) | 1.29±0.03 | 8.38±0.05 | 9.35±0.04 |
| Organic matter (SOM; g kg^-1^) | 22.2±0.2 | 144.4±1.4 | 161.2±1.6 |
| C/N ratio | 13.16±0.5 | 6.27±0.3 | 7.14±0.4 |
| Cation exchange capacity (CEC; cmole_c_/kg soil) | 33.9±0.4 | 36.3±0.5 | na |
| Available-N (mg/kg) | 42±2.1 | 532±22.2 | 490±19.6 |
| Available-P (mg/kg) | 7±0.05 | 54±0.9 | 151±2.4 |
| Available-K (mg/kg) | 103±1.9 | 66±0.9 | 515±2.5 |
| Total-N (g/kg) | 0.98±0.04 | 13.37±0.81 | 13.09±0.85 |
| Total-P (mg/kg) | 133±21 | 444±24 | 833±28 |
| DTPA-Cu (mg/kg)^¥^ | 0.92±0.03 | 24.80±0.91 | 4.90±0.15 |
| DTPA-Ni (mg/kg) | 0.20±0.01 | 2.80±0.05 | 2.80±0.03 |
| DTPA-Pb (mg/kg) | 0.70±0.02 | 14.00±0.08 | 3.60±0.05 |
| Sand (%) | 18.20±0.23 | na^†^ | na |
| Silt (%) | 57.54±1.21 | na | na |
| Clay (%) | 24.26±0.98 | na | na |
| Texture class | Silty loam | na | na |
| Total bacterial count (CFU^⸸^ 10^6^/g soil) | 50.0±1.21 | 133.0±2.33 | 3.0±0.02 |
| Total fungi count (CFU 10^6^/g soil) | 1.6±0.01 | 5.0±0.02 | 0.9±0.01 |
| Total actinomycetes count (CFU 10^6^/g soil) | 3.3±0.1 | 10.6±0.3 | 1.8±0.1 |
| Spore-forming bacteria count (CFU 10^6^/g soil) | 0.5±0.01 | 0.6±0.01 | 0.2±0.01 |
| *Azotobacter* count (CFU 10^4^/g soil) | 8.8±0.14 | nd^‡^ | nd |
| *Azospirillium* count (CFU 10^4^/g soil) | 2.1±0.10 | nd | nd |
| Phosphatase activity (mg p-nitrophenol/g soil/h) | 35.5±0.21 | nd | nd |
| Dehydrogenase activity ( mg TPF/kg soil/day)^**^ | 6.91±0.18 | nd | nd |
| Catalase activity (µmole H_2_O_2_/g soil/15 min) | 187±13 | nd | nd |
| Invertase activity (µmole glucose/g soil/day) | 5.78±0.11 | nd | nd |
| ^*^ pH was measured in (1:2.5 W/V soil:water suspension and 1:10 biosolids:water suspension); ^†^ not available; ^‡^ not detected; ^¥^ Diethylenetriaminepentaacetic acid; ^⸸^ cell forming unit; ^**^ triphenylformazan | | | |
